# LipG a bifunctional phospholipase/thioesterase involved in mycobacterial envelope remodeling

**DOI:** 10.1042/BSR20181953

**Published:** 2018-12-18

**Authors:** Pierre Santucci, Vanessa Point, Isabelle Poncin, Alexandre Guy, Céline Crauste, Carole Serveau-Avesque, Jean Marie Galano, Chistopher D. Spilling, Jean-François Cavalier, Stéphane Canaan

**Affiliations:** 1 Aix-Marseille Univ, CNRS, LISM, IMM FR3479, Marseille, France; 2 Institut des Biomolécules Max Mousseron (IBMM), UMR 5247, Université de Montpellier, CNRS, ENSCM, 15 Avenue Charles Flahault, 34093 Montpellier Cedex 5, France; 3 Aix-Marseille Univ, CNRS, Centrale Marseille, ISM2, Marseille, France; 4 Department of Chemistry and Biochemistry, University of Missouri, One University Boulevard, St. Louis, MO 63121, U.S.A.

**Keywords:** antibiotics, cell-envelope, lipolytic enzymes, Mycobacterium tuberculosis, phospholipid homeostasis

## Abstract

Tuberculosis caused by *Mycobacterium tuberculosis* is currently one of the leading causes of death from an infectious agent. The main difficulties encountered in eradicating this bacteria are mainly related to **(i)** a very complex lipid composition of the bacillus cell wall, **(ii)** its ability to hide from the immune system inside the granulomas, and **(iii)** the increasing number of resistant strains. In this context, we were interested in the *Rv0646c* (*lipG_MTB_*) gene located upstream to the *mmaA* cluster which is described as being crucial for the production of cell wall components and required for the bacilli adaptation and survival in mouse macrophages*.* Using biochemical experiments combined with the construction of deletion and overexpression mutant strains in *Mycobacterium smegmatis*, we found that LipG_MTB_ is a cytoplasmic membrane-associated enzyme that displays both phospholipase and thioesterase activities. Overproduction of LipG_MTB_ decreases the glycopeptidolipids (GPL) level concomitantly to an increase in phosphatidylinositol (PI) which is the precursor of the PI mannoside (PIM), an essential lipid component of the bacterial cell wall. Conversely, deletion of the *lipG_MS_* gene in *M. smegmatis* leads to an overproduction of GPL, and subsequently decreases the strain susceptibility to various antibiotics. All these findings demonstrate that LipG is involved in cell envelope biosynthesis/remodeling, and consequently this enzyme may thus play an important role in mycobacterial physiology.

## Introduction

With more than 10 million new cases and almost 1.6 million deaths in 2017, tuberculosis (TB) caused by the etiologic agent *Mycobacterium tuberculosis* (*M. tuberculosis*) still remains the deadliest infectious disease worldwide [1]. The current antibiotherapy, which is a combination of four antibiotics (i.e. isoniazid (INH), rifampicin (RIF), pyrazinamide, and ethambutol), allows to heal 95% of infected people with active disease when administered under directly observed therapy [2]. This treatment is, however, not effective enough to overcome the emergence of multidrug and extensively drug-resistant strains of *M. tuberculosis* [3,4]. More recently, some totally drug-resistant *M. tuberculosis* strains have also been detected, which are virtually incurable [5]. Most of the time this antibiotic resistance is directly related to the unique mycobacterial cell wall composition which is an essential factor contributing to virulence, pathogenesis, and survival of the bacilli. Therefore, identifying proteins and mechanisms involved in the biosynthesis and/or remodeling of this complex structure could lead to the development of new therapeutics to control and fight TB.

Indeed, amongst all bacterial phyla, *Actinobacteria* and more specifically *M. tuberculosis*, possess an unusual cell envelope with a specific architecture constituted by lipids which may represent up to 40–60% of the dry weight of the cell, as compared with only 5–10% in other bacteria [6,7]. The outer membrane is an extremely hydrophobic barrier mainly composed of long-chain mycolic acids (MAs) and several atypical lipids, which are responsible for the high intrinsic tolerance of the bacteria to various antibiotics [8]. Moreover, it is now acknowledged that most of these lipids play important roles in the viability and/or virulence of the bacteria [9]. As a consequence, the structural characterization of these complex lipids, as well as their biosynthetic pathways have been the subject of many studies in the past decade with the aim of identifying putative therapeutic targets [10–12].

Of particular interest, the synthesis of MAs involves two fatty acid synthase (FAS) enzymatic complexes, FAS-I and FAS-II [13,14]. FAS-I synthesizes *de novo* medium chain fatty acids (C16–18 and C24–26) as acyl-CoA derivatives, which are further elongated by the FAS-II complex to form the C48–64 meromycolic chains, a major component of the envelope arabinogalactan layer [13,14]. The various methyltransferases closely interact with enzymes belonging to the FAS-II system to functionalize meromycolate chains by adding specific chemical modifications (such as cyclopropane, methyl, hydroxyl, and ketone) [14–18]. Amongst the eight putative methyltransferases identified in *M. tuberculosis* H_37_Rv genome, the *mmaA* gene cluster; i.e. *mmaA1* (*Rv0645c*), *mmaA2* (*Rv0644c*), *mmaA3* (*Rv0643c*), and *mmaA4* (*Rv0642c*); is essential for chemical group introduction and functionalization of Mas, thus participating actively in the envelope integrity/virulence of *M. tuberculosis* [14,19,20].

Interestingly, these *mmaA* genes are also clustered with the *Rv0646c* gene, which is highly conserved amongst mycobacterial species and encodes for LipG (LipG_MTB_), a putative lipase/esterase which has been recently described as a carboxylesterase [21]. *M. tuberculosis* H_37_Rv genome encodes for 36 lipolytic enzymes annotated as putative esterases, lipases, cutinases, or phospholipases based on their sequence homology and the presence of the consensus G-x-S-x-G motif, characteristic of the α/β hydrolase-fold family [19,22–24]. LipG_MTB_ has been classified within the Lip family which includes 24 members [23,25]. This enzyme possesses approximately 50% sequence identity with the EstB protein from *Acinetobacter calcoaceticus* and 43% with the putative hydrolase PA3586 from *Pseudomonas aeruginosa* PAO1. Interestingly, the *Rv0646c* gene has been annotated as essential for *M. tuberculosis* H_37_Rv survival within primary murine cells by transposon site hybridization [26]. Due to its high conservation and its peculiar location upstream to the *mmaA* cluster, which is crucial for the production of cell wall components and so for the mycobacterial envelope integrity, the potential physiological function of LipG deserves to be studied and deciphered.

In this context, *Rv0646c* gene from *M. tuberculosis* H_37_Rv has been cloned, expressed, purified, and fully biochemically characterized. Since its potential important role in mycobacterial lifecycle makes LipG_MTB_ an attractive target for future therapeutic developments against TB, inhibition studies on pure recombinant enzyme were also conducted with two recent families of antitubercular compounds; namely, oxadiazolone-core (**OX**) derivatives [23,27], and monocyclic analogs of Cyclophostin and Cyclipostins analogs (**CyC**) [28–30] which affect the growth of *M. tuberculosis* both *in vitro* and in infected macrophages.

In order to investigate the physiological function of LipG in mycobacterial species, both deletion and overexpression mutant strains have been generated into the surrogate strain *Mycobacterium smegmatis*. By using phenotypic assays, subcellular location, *in silico* modeling, lipid extraction followed by TLC analysis, and susceptibility testing via the resazurin microtiter assay (REMA), we determined that LipG_MTB_ is a cytoplasmic membrane-associated enzyme involved in phospholipids remodeling and cell wall integrity.

## Experimental procedures

### Bacterial strains and culture conditions

*Escherichia coli* DH10β and *E. coli* C41 (DE3)/pLyS strain were cultured in LB broth or Terrific Broth (Invitrogen, France). *M. smegmatis* mc^2^ 155 and *M. smegmatis* mc^2^ 155 *groEL1ΔC* [31] strains were cultured in Middlebrook 7H9 broth (BD Difco, Le Pont-de-Claix, France) supplemented with 0.05% (*v/v*) Tween-80 and 0.2% (*v/v*) glycerol (Sigma–Aldrich, Saint-Quentin Fallavier, France) (7H9-S). When needed, ampicillin and kanamycin (Euromedex, Souffelweyersheim, France) were added to the medium at final concentrations of 100 and 50 µg/ml for both *E. coli* and mycobacterial species, respectively. Hygromycin B (Euromedex) was used at a final concentration of 200 and 50 µg/ml for recombinant *E. coli* and recombinant mycobacteria, respectively.

### *In silico* analyses

Genomic sequences of *lipG* and its orthologs were obtained from the NCBI database. *M. tuberculosis* H37Rv, *Mycobacterium leprae* TN, *M. marinum* M, and *M. smegmatis* genomic clusters were re-adjusted manually based on the genomic annotations provided by the Mycobrowser database [32]. The amino acid sequence of *M. tuberculosis* H_37_Rv LipG and its orthologs were aligned using Multalign software [33] and results were displayed using ESPrit 3.0 software [34]. The percentage of identity between the different orthologs was generated with the Clustal Ω multiple sequence alignment program (http://www.ebi.ac.uk/Tools/msa/clustalo/). A 3D model of *M. tuberculosis* H_37_Rv LipG (LipG_MTB_) was also generated, by using the I-TASSER server [35,36] and visualized with PyMOL software (The PyMOL Molecular Graphics System, version 1.8 Schrödinger, LLC). Electrostatic surface potentials of LipG_MTB_ model structure was built using freely available PDB2PQR web service [37,38]. The electrostatic potential data thus generated, were further visualized using the PyMOL APBS Tools 2 plugin [39].

### Construction of recombinant plasmids, expression and purification of recombinant LipG_MTB_ protein

*Rv0646c* ORF encoding LipG_MTB_ protein, was amplified from *M. tuberculosis* H_37_Rv genomic DNA by polymerization chain reaction, using primers containing the attB1, Shine–Dalgarno, 6× His, and a TEV (tobacco etch virus) NIa site sequences at the 5′-end and the attB2 recombination site at the 3′-end (Supplementary Table S1, P1_MTB_ and P2_MTB_). The resulting PCR fragments were cloned using the Gateway® technology (Invitrogen, Carlsbad, CA, U.S.A.) into the pDonR221 entry vector, and then into the pDEST14 expression vector (pDEST14*::lipG_MTB_*). A similar strategy was used to clone the *MSMEG_1352* ORF encoding LipG_MS_ protein. PCR was performed with P1_MS_ and P2_MS_ primers (Supplementary Table S1), thus giving the pDEST14::*lipG_MS_* construct. DNA sequences of recombinant clones were analyzed by GATC Biotech (Ebersberg, Germany). The newly generated pDEST14*::lipG_MTB_* was used as a template for generating pDEST14*::lipG_MTB_^S123A^*by PCR mutagenesis using specific primers (Supplementary Table S1, P3 and P4). The resulting plasmids pDEST14*::lipG_MTB_*, pDEST14*::lipG_MTB_^S123A^*, and pDEST14::*lipG_MS_* were used to transform *E. coli* C41 (DE3)/pLyS strain harboring the pRare plasmid (Novagen).

For protein expression, 1 L of Terrific Broth medium was inoculated with the recombinant strains at initial OD_600_ of 0.05, and was further incubated at 37°C and 200 rpm. When the OD_600_ reached 0.6, cells were put on ice for 30 min. Then, 0.5 mM isopropyl-d-thiogalactopyranoside was added to the medium and the culture was further incubated at 17°C under shaking. After 18 h of incubation, cells were pelleted, re-suspended in 30 ml of ice-cold lysis buffer (50 mM Tris/HCl pH 8.0, 150 mM NaCl, 1 mM EDTA, 0.1% (*v/v*) Triton X-100, 0.25 mg/ml lysozyme), and stored at −80°C. To purify recombinant LipG_MTB_ or LipG_MS_, the supernatant (S1) obtained after cell lysis and centrifugation at 17000×***g*** for 30 min, was stored at 4°C. Pellet was then re-suspended in lysis buffer, sonicated twice for 30 s with 30 s breaks between each cycle, solubilized by stirring at 4°C for 1 h using a magnetic bar, and further centrifuged at 17000×***g*** for 30 min (giving S2 supernatant). Both supernatants (S1 and S2) were pooled and loaded on to a Ni^2+^-NTA affinity column previously equilibrated with buffer A (10 mM Tris/HCl pH 8.0, 150 mM NaCl). The column was washed with buffer A before performing elution with buffer A and 125 and 250 mM of imidazole. Fraction purity was assessed on SDS/PAGE. The corresponding 35 kDa protein band was further analyzed by tryptic digestion and MS. Pure protein was concentrated to 0.5–1 mg/ml, flash frozen in liquid nitrogen, and stored at −80°C.

## Biochemical characterization and inhibition of rLipG_MTB/MS_

### Esterase activity assay

The esterase activity of rLipG_MTB_ or rLipG_MS_ was determined as described previously [23] with slight modifications. Briefly, *p*-nitrophenyl (*p*NP) esters with different carbon chain lengths were used as substrates, including *p*NP acetate (*p*NP-C2), butyrate (*p*NP-C4), caproate (*p*NP-C6), caprylate (*p*NP-C8), caprate (*p*NP-C10), laurate (*p*NP-C12), myristate (*p*NP-C14), and palmitate (*p*NP-C16). Stock solution (200 mM) of each substrate was freshly prepared in acetonitrile. Release of *p*NP was monitored at 410 nm and pH 8 using a 96-well plate spectrophotometer (PowerWave™, Bio-Tek Instruments) and quantitated using a *p*NP calibration curve (10 µM to 0.5 mM) with _(λ = 410 nm)_ = 6.0 mM^−1^. Enzymatic reactions were performed at 37°C over a period of 20 min in a 96-well microplate filled with 10 mM Tris buffer (pH 8.0) containing 150 mM NaCl with 0.5% (*v/v*) Triton X-100 to a final volume of 200 µl. In each well, 2 mM of substrate and 1 µg of rLipG_MTB_ (0.15 µM final concentration) were added. Activities were expressed in international units (U), corresponding to 1 µmol of *p*NP released per min. Specific activities (S.A) were expressed as U/mg of pure enzyme. Experiments were done in triplicate.

### Phospholipase A_1_ and A_2_ activities assay

The phospholipase A_1_ and A_2_ activities of rLipG_MTB_ and rLipG_MS_ were monitored continuously using BODIPYH dye-labeled phospholipids: PED-A_1_ (N-((6-(2,4-DNP)Amino)Hexanoyl)-1-(BODIPYH FL C5)-2-hexyl-*sn*-glycero-3-phosphoethanolamine) and red/green BODIPYH PC-A_2_(1-*O*-(6-BODIPYH558/568-aminohexyl)-2-BODIPYH FLC5-*sn*-glycero-3-phosphocholine), respectively, as previously described [40,41]. Briefly, substrate stock solutions (50 µM) were prepared in ethanol. All enzyme activities were assayed in 10 mM Tris (pH 8.0), 150 mM NaCl. Enzymatic reactions were performed at 25°C for 60 min in a final volume of 200 µl containing 1.5 µg rLipG_MTB_ (0.23 µM final concentration) and 5 µM substrate. The release of BODIPYH (BFCL5) (Life Technologies) was recorded at λ_exc_ = 485 nm and λ_em_ = 538 nm using a 96-well plate fluorimeter (Fluoroskan Ascent, Thermo Fisher Scientific). Each experiment was done in triplicate. Enzymatic activities were quantitated using a BFCL5 calibration curve (0.08–200 pmoles). S.A were expressed in mU of fatty acid (or BFLC5) released per mg of pure protein (mU.mg^−1^). One unit correspond to the release of one µmol of product release per min. PLA_1_ from *T. lanuginosus* and Bv PLA_2_ (Sigma–Aldrich) were used as positive standards for the PLA_1_ and the PLA_2_ activities, respectively.

### Thioesterase activity assay

Hydrolysis of palmitoyl-Coenzyme A (palmitoyl-CoA) (Sigma–Aldrich) was used to measure thioesterase activity. After the hydrolysis of the thioester bond, the free sulphur on CoA is attacked by DTNB (5-5′-Dithio-bis 2-nitrobenzoic acid) (Sigma–Aldrich) which releases a measurable nitrophenyl group, TNB^2−^ (5-Thio-2-nitrobenzoate) ion detectable at 415 nm. Fresh stock solution of palmitoyl-CoA (1 mM) and DTNB (0.4 mM) were prepared in water. Palmitoyl-CoA and DTNB were added in each well of a 96-well plate at final concentration of 10 µM. The thioesterase activity of rLipG_MTB_ or rLipG_MS_ (30 µg) was compared with that of TesA (Rv2928) [42] and LipY [43] used as positive and negative controls, respectively. Triplicate assays were done at 37°C in 10 mM Tris (pH 8.0) 150 mM NaCl buffer. The absorbance at 415 nm was continuously measured and the release of TNB^2−^ was quantitated using a calibration curve (0.5–30 nmoles).

### Enzymatic inhibition assays using OX derivatives, CyC analogs, and Orlistat

The six oxadiazolone derivatives (**OX**); i.e. **M*m*PPOX, iBPOX, iB*p*PPOX, HPOX, H*p*PPOX**, and **BePOX**; were synthesized as described previously [23,27]. The five **CyC** analogs; i.e. **CyC_17_, CyC_7α_, CyC_7β_, CyC_8α_**, and **CyC_8β_**; were synthesized according to [44–46].

Inhibition experiments were performed using the classical enzyme-inhibitor pre-incubation method, as described previously [30,47,48]. Stock solutions (10 mM) of each **CyC** and **OX** inhibitor, as well as **Orlistat** were prepared in DMSO. rLipG_MTB_ was pre-incubated for 30 min at 25°C with each compound at various inhibitor molar excess (*x*_I_), related to 1 mol of enzyme, ranging from 1 to 200. Sample of the incubation medium (1.0 µg rLipG_MTB_) was then collected for measuring the enzyme residual activity using the colorimetric assay with the substrate *p*NP-C6, as described above. The variation in the residual activity allowed to determine the inhibitor molar excess which reduced the enzyme activity to 50% of its initial value (*x*_I50_) [47,48]. Thereby, a *x*_I50_ value of 0.5 is corresponding to a 1:1 stoichiometric ratio between the enzyme and the inhibitor, and is therefore the highest level of inhibitory activity that can be achieved. In each case, control experiments were done in the absence of inhibitor but with the same volume of solvent. It is worth noting that DMSO at a final volume concentration of less than 10% has no effect on the enzyme activity. Inhibition dose–response curves were fitted in Kaleidagraph 4.2 Software (Synergy Software). Results are expressed as mean values ± S.D. of at least three independent assays.

### Creation of *M. smegmatis MSMEG_1352* disrupted strain

The *MSMEG_1352* gene encoding LipG_MS_ was amplified with 500-bp flanking regions by PCR using *M. smegmatis* mc^2^ 155 genomic DNA as template and specific primers (Supplementary Table S1, P9 and P10). PCR product of ∼2000 bp was cloned into pJET1.2/blunt vector following manufacturer’s instructions giving pJET*-lipG_MS_*. Recombinant plasmid was then digested with NruI, and a hygromycin resistance cassette amplified from pUC-Hygro using primer (Supplementary Table S1, P17 and P18). The resulting blunt-ended PCR product was cloned in order to disrupt *lipG_MS_* gene. This new plasmid, pJET*-lipG_MS_::Hyg* which contains an inactive version of *lipG_MS_* gene was used as template for amplifying an allelic exchange substrate by using specific primers (Supplementary Table S1, P9 and P10). Approximately 300 ng of purified PCR product was electroporated into electro-competent *M. smegmatis* mc^2^ 155 strain harboring pJV53 plasmid, and cells were plated on to Middlebrook 7H10 agar medium supplemented with 50 µg/ml of both kanamycin and hygromycin and incubated at 37°C during 5 days as previously described [49]. Finally, recombinant clones were analyzed by PCR using P7-P8 and P11-P12 primers’ pairs (Supplementary Table S1).

### Creation of overexpression/complementation vectors

ORFs *Rv0646c* and *MSMEG_1352* were amplified by PCR with specific primers (Supplementary Table S1, P5–P6 and P7–P8) containing NdeI and BamHI restriction sites in 5′ and 3′ ends, respectively. Digested and purified products were cloned into the pVV16 mycobacterial expression vector in frame with the _6_His-tag located in C-terminal position giving the pVV16::*lipG_MTB_* and pVV16::*lipG_MS_* complementation plasmids allowing a constitutive production of recombinant LipG. *Rv0646c* ORFs was also cloned within a homemade pMyC Gateway® vector, allowing a strong overexpression following acetamide induction, thus giving pMyC::*lipG_MTB_*. Competent *M. smegmatis* mc^2^ 155, *M. smegmatis groEL1ΔC*, and *lipG_MS_ ::Hyg* cells were prepared as previously described [50] and were electroporated with a single pulse of 2.5 kV, 25 µF, and 1000 Ω by using a Gene Pulser Xcell™ Electroporation System (Bio-Rad). Selection was achieved by plating cells on complete Middlebrook agar medium supplemented with the proper antibiotic.

### Subcellular fractionation and immunoblotting

Recombinant *M. smegmatis groEL1ΔC* harboring pVV16::*lipG_MTB_* and pVV16::*lipG_MS_* were cultured in 7H9-S medium supplemented with 50 µg/ml kanamycin until OD_600_ was approximately 1–1.5. Cells were harvested for 10 min at 4000×***g***, then washed twice with PBS (pH 7.4) buffer containing 0.05% (*v/v*) Tween-20, and re-suspended in lysis buffer (PBS pH 7.4 containing EDTA and 1 mM PMSF). Cells were then disrupted by using a French pressure cell at 1100 *psi*. Subcellular fractionation was performed as previously described [51] with slight modifications. Briefly, the cell lysate was centrifuged two times at 1000×***g*** at 4°C in order to remove unbroken cells. Supernatant (total lysate) was further centrifuged two times at 27000×***g*** at 4°C to separate cell wall/membrane from the cytoplasmic fraction. The pellet containing insoluble material was washed twice with PBS (pH 7.4) to remove any cytoplasmic contaminant, and supernatant containing the cytoplasmic fraction was further ultracentrifuged at 100000×***g*** for 90 min at 4°C to remove any membrane contaminant. Each fraction was loaded and separated on to 12% SDS/PAGE and transferred on to a nitrocellulose membrane using a Trans-Blot Turbo Transfer System (Bio-Rad). Immunoblotting of 6× His-tagged proteins was performed using anti-HisProbe™ HRP conjugate (Thermo Fisher Scientific). MSMEG_0220 was used as the control for subcellular location, and immunoblotting was performed by using rabbit polyclonal antibodies [52,53] and horseradish peroxidase–conjugated anti-rabbit IgG (Sigma-Aldrich). Revelation was achieved using Pierce™ ECL Western Blotting substrate solution (Thermo Fisher Scientific) and visualized with a ChemiDoc™ MP Imaging System (Bio-Rad).

### Genapol® extraction and immunoblotting

Approximately 10 uOD_600_ of recombinant cells cultured in 7H9-S medium supplemented with 50 µg/ml kanamycin at OD_600_ ∼1-1.5 were harvested for 10 min at 4000×***g***. Cells were washed twice in PBS (pH 7.4) buffer containing 0.05% (*v/v*) Tween-20 and surface-exposed proteins were isolated by incubating cells with PBS containing 0.5% (*v/v*) Genapol®-X080 (Sigma–Aldrich) at room temperature [54,55]. Control samples were treated with a PBS buffer without Genapol®-X080. After approximately 30 min, all supernatants were collected and precipitated with 12% (*v/v*) trichloroacetic acid. Finally, samples were separated on to 12% SDS/PAGE and immunoblotting was achieved as described above.

### Dynamic subcellular location using fluorescence microscopy

Gene encoding superfolder GFP (sfGFP) was amplified from pKD4-*sfGFP* plasmid with specific primers (Supplementary Table S1, P13–P15 and P14–P15) which were cloned in pMV261 and pVV16 vectors at either EcoRI/HindIII or only HindIII restriction sites. Thus, creating pMV261::*sfGFP* and pVV16::*sfGFP. Rv0646c* and *MSMEG_1352* ORFs were amplified by PCR with specific primers (Supplementary Table S1, P5–P6 and P7–P8) containing NdeI and BamHI restriction sites in 5′ and 3′ ends, respectively. Digested and purified products were cloned into the pVV16::*sfGFP* in frame with the *sfGFP* gene and thus generating a translational fusion. *M. smegmatis* was electroporated with 1.5 µg of pMV261::*sfGFP* and these latter constructs; recombinant clones were selected on to specific medium and overexpression of the two chimeric proteins were checked by immunoblotting as described above. Recombinant strains were cultured until OD_600_ reaches ∼1-1.5, and then harvested by centrifugation at 4000×***g*** for 10 min. Cells were washed once in PBS (pH 7.4) buffer, and bacterial suspension (5 µl) was spotted between a coverslip of 170 µm thickness and a 1.5% agarose-PBS pad. Bacteria were analyzed by snapshot imaging at room temperature using an Olympus FV1000 confocal microscope and 100× oil-objective. Exposure time was 800 ms for both phase-contrast and fluorescence images (λ_exc_\λ_em_ = 490/530 ±10 nm). Images recorded were processed using the open source program ImageJ 1.51K (NIH, U.S.A.).

### Lipid extraction and analysis

Mycobacterial strains were grown in 1 l of 7H9-S broth until an OD_600_ of ∼1 was reached, except when using acetamide inducible strains where cells were collected at specific time points. Cultures were then centrifuged at 4000***g*** for 15 min at 4°C and washed twice with distilled water. Pellets were lyophilized overnight and weighed to determine the exact mass of bacterial dry extract for normalization calculations.

MAs methyl esters (MAMEs) and fatty acids methyl esters (FAMEs) were extracted following previously described protocol [56,57]. Approximately, 10 mg of cellular dry weight were re-suspended in 2 ml TBAH (40% *v/v*, Sigma–Aldrich) and incubated at 100°C overnight. Then 4 ml of CH_2_Cl_2_, 300 µl CH_3_I, and 2 ml of water were added to the mixture and incubated for 1 h at room temperature under shaking. Sample was centrifuged and the lower organic phase was kept, washed three times with distilled water, dried over MgSO_4_, and then concentrated under nitrogen stream. Finally, 3 ml of DTT were added to the dried extract, and sonication was performed for 10 min in a water bath. Sample was centrifuged for 10 min at 4000×***g*** to remove any precipitate. The DTT layer was evaporated and the residue was re-suspended in 100 µl of DTT. The same volume of extract containing MAMEs and FAMEs were separated on 1D TLC using hexane/ethyl acetate (19:1, *v/v*) as eluent, and revealed by using a 10% phosphomolybdic acid solution in absolute ethanol.

Total lipids were extracted as previously described [58] with slight modifications. Briefly, lipids from dry extract were incubated for 16 h with CHCl_3_-MeOH (1:2; *v/v*), at room temperature under shaking. Residual lipids were re-extracted for 16 h with the same solvents, but using alternative ratios (1:1 and 2:1 *v/v*). All the three organic phases were pooled and concentrated under reduced pressure. Samples were re-suspended in a CHCl_3_-MeOH solution (3:1, *v/v*), and washed with 0.3% (*w/v*) NaCl in water. Organic and aqueous phases were separated by centrifugation 4000×***g*** for 10 min, and only the organic phases were conserved and dried over MgSO_4_. Samples were finally evaporated under a nitrogen stream, weighed, and re-suspended in a CHCl_3_-MeOH solution (3:1, *v/v*).

Equal volume of extract containing glycopeptidolipids (GPLs) were separated on TLC (Silica Gel 60, Merck) by using CHCl_3_-MeOH (90:10, *v/v*) as eluent and further visualized by vaporization of 20% (*v/v*) sulphuric acid in ethanol. Phospholipid analyses were carried out by using both 1- or 2D TLC. Mono-dimensional TLC were performed by loading the same volume of extract and using CHCl_3_-MeOH-H_2_O (65:25:4; *v/v/v*) as eluent. Concerning bi-dimensional TLC, the first dimension was resolved by using CHCl_3_-MeOH-H_2_O (65:25:4; *v/v/v*) as solvent system 1, and CHCl_3_-MeOH-CH_3_COOH-H_2_O (80:12:15:3; *v/v/v/v*) as solvent system 2. Phospholipids were visualized by vaporization of a copper acetate/orthophosphoric acid mixture prepared by mixing a copper acetate saturated solution with a 85% (1:1, *v/v*) aqueous orthophosphoric acid solution. Phospholipid standards were purchased from Sigma–Aldrich. Finally, each resolved plate was heated at 120°C for 5–10 min, scanned using a ChemiDoc™ MP Imaging System (Bio-Rad), and densitometric analyses was performed using the ImageLab™ software version 5.0 (Bio-Rad) allowing to determine the relative content of each sample.

###  REMA for MIC determination

Susceptibility testing was performed using the Middlebrook 7H9 broth microdilution method. All assays for each strain were carried out at least in triplicate. MICs against the various *M. smegmatis* bacterial strains were determined in 96-well flat-bottom Nunclon Delta Surface microplates with lid (Thermo Fisher Scientific) using REMA method [59]. Briefly, log-phase bacteria were diluted to a cell density of 5 × 10^6^ cells/ml in 7H9-S medium. Then 100 µl of the above inoculum (i.e. 5 × 10^5^ cells per well) was added to each well containing 100 µl of 7H9-S medium, serial two-fold dilutions of the selected antibiotics (RIF or INH), **CyC_17_** [28,29], **HPOX** [27], or **Orlistat** to a final volume of 200 µl. Growth controls containing no inhibitor (*i.e.*, bacteria only), inhibition controls containing 50 μg/ml kanamycin and sterility controls (i.e. medium only) without inoculation were also included. Plates were incubated at 37°C in a humidity chamber [60] to prevent evaporation for 3–5 days. Then, 20 µl of a 0.02% (*w/v*) resazurin solution was added to each well, and the plates were incubated at 37°C for color change from blue to pink or violet and for a reading of fluorescence units (FU). Fluorescence corresponding to the resazurin reduction to its metabolite resorufin was quantitated using a Tecan Spark 10M multimode microplate reader (Tecan Group Ltd, France) with excitation at 530 nm and emission at 590 nm. Fluorometric MICs were determined by fitting the RFU% sigmoidal dose–response curves [28,29] in Kaleidagraph 4.2 software (Synergy Software). MIC values determined as the lowest compound concentrations inhibiting 50% of growth were defined as the MIC_50_.

## Results

### LipG is a highly conserved mycobacterial protein

**Figure 1:**
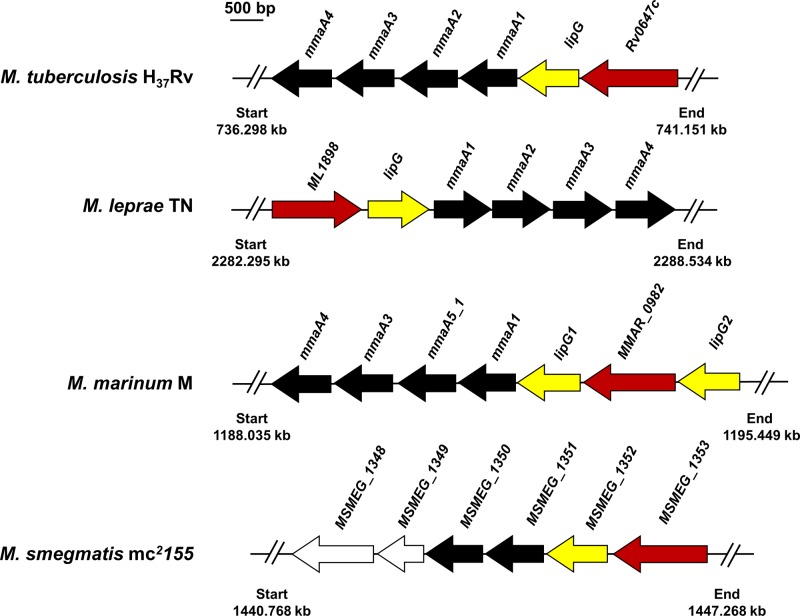
Genomic organization of *lipG* clusters in mycobacterial species Schematic organization of the *lipG* genomic loci from *M. tuberculosis, M. leprae, M. marinum*, and *M. smegmatis* were designed based on the Mycobrowser database [32]. *lipG* genes are represented in yellow whereas genes involved in MAs maturation processes are in black. Furthermore, a conserved ORF encoding a putative ABC transporter was highlighted in red. Co-ordinates of each gene cluster are indicated in kb. Scale represents 500 bp.

Gene *Rv0646c* encoding LipG_MTB_ protein was not only found within *M. tuberculosis* genome. Genomic context containing *Rv0646c* gene and its orthologs was indeed surprisingly highly conserved within many mycobacterial species. In *M. tuberculosis, Rv0646c* is surrounded by an essential uncharacterized gene (*Rv0647c*) and the *mmaA* cluster composed of four *mmaA* genes (*mmaA1–4*) encoding proteins involved in MAs maturation processes (Figure 1). Interestingly, the occurrence and genome organization of *lipG* clusters are also highly conserved in *M. tuberculosis, M. leprae, M. marinum*, or *M. smegmatis* genomes (Figure 1), with two copies of genes encoding for LipG proteins in *M. marinum, M. abscessus*, or *M. ulcerans* genomes. Multiple sequence alignment of these various LipG proteins and sequence comparison, revealed a high level of amino-acid conservation, also with some consensus amino-acids blocks between each mycobacterial species [21] (Supplementary Figure S1). The LipG characteristics (i.e. sizes of genes/proteins and sequence identities) from different mycobacterial strains are summarized in Table 1. Each of these proteins possess a molecular weight ∼32 kDa, with the number of amino acids ranging from 301 to 324. Taken together, all these findings suggest that the cluster including the *lipG* gene may be dedicated to a common mycobacterial feature either in strict pathogenic, opportunistic, or non-pathogenic strains.

**Table 1:** Conservation and characteristics of mycobacterial LipG proteins

Mycobacterial species/strains	LipG and orthologs				
	Gene	Gene ID	Protein	Protein size (aa)	Identity^1^ (%)
*M. tuberculosis* H_37_Rv	*Rv0646c*	*888065*	LipG	301	-
*M. bovis* AF2122/97	*Mb_0665c*	*1091775*	LipG	301	100
*M. bovis* BCG Pasteur 1173P2	*BCG_0695c*	*4697156*	LipG	301	100
*M. canetti* CIPT 140010059	*MCAN_06451*	*10986452*	LipG	301	99
*M. leprae* TN	*ML_1899*	*910591*	LipG	305	71
*M. marinum* M	*MMAR_0981*	*6225233*	LipG_1_	315	73
	*MMAR_0983*	*6225235*	LipG_2_	310	47
*M. ulcerans* Agy99	*MUL_0733*	*4552132*	LipG_1_	314	73
	*MUL_0735*	*4552134*	LipG_2_	303	47
*M. abscessus* ATCC 19977	*MABS_3878*	*5967421*	LipG_2_	310	40
	*MABS_3880*	*5966346*	LipG_1_	324	54
*M. smegmatis* mc^2^155	*MSMEG_1352*	*4534086*	LipG	305	64

1 Amino-acid sequence identity related to *M. tuberculosis* H37Rv.

### Cloning, expression purification, and biochemical characterization of LipG_MTB_

**Figure 2:**
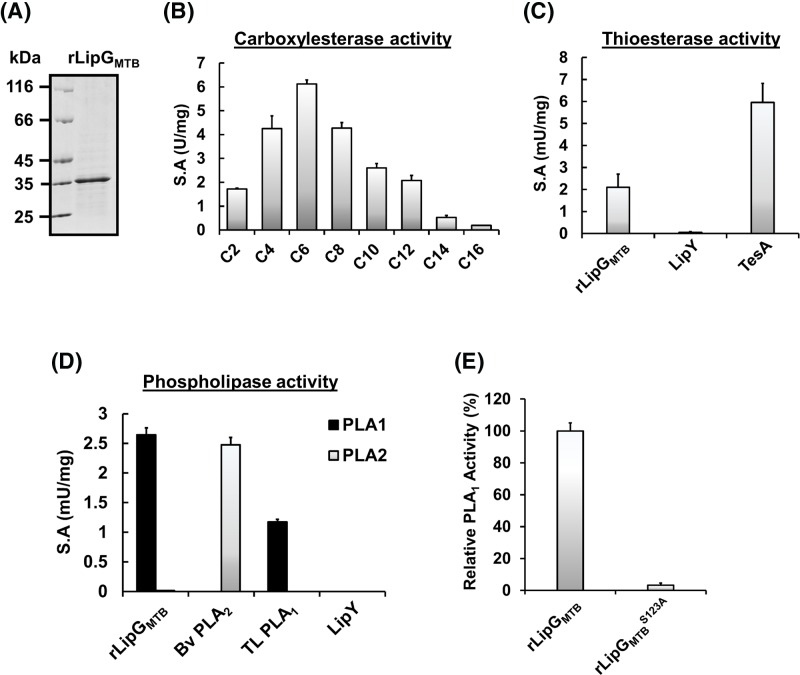
Purification and biochemical characterization of recombinant rLipG_MTB_ (**A**) Purity assessment of rLipG_MTB_. rLipG_MTB_ (5 µg) was loaded on to 12% polyacrylamide gel. Unstained protein molecular weight marker was used as standard (Euromedex). (**B**) Carboxylesterase S.A determination on *p*NP-ester substrates with chain lengths varying from C2 to C16. (**C**) Thioesterase activity measurements corresponding to palmitoyl-CoA hydrolysis. (**D**) PLA_1_ and PLA_2_ activity using fluorescent phospholipids. TL PLA_1_, Bv PLA_2_, and LipY were used as positive and negative controls, respectively. (**E**) PLA_1_ enzymatic activity of the rLipG_MTB_^S123A^ compared with rLipG_MTB_. Results are expressed as relative activity in %. Each result is expressed as mean values ± S.D. of at least three independent assays. Abbreviation: Bv PLA_2_, *Bee venom* PLA_2_, TL PLA_1_, *T. lanuginosus* lipase PLA_1_.

In an attempt to characterize LipG biochemical activities more finely, to confirm its carboxylesterase properties [21], and also to decipher the physiological role of this protein, the *Rv0646c* and *MSEG_1352* genes have been cloned. The respective recombinant rLipG_MTB_ and rLipG_MS_ were produced in *E. coli* C41 (DE3)/pLyS strain harboring the pRare plasmid. rLipG_MTB_ and purification was achieved using Ni^2+^-NTA affinity chromatography, yielding ∼1 mg of pure recombinant protein per culture liter. The purity and the expected molecular weight (∼35 kDa) were confirmed by 12% SDS/PAGE (Figure 2A) and MALDI-TOF analysis.

The esterase activity of rLipG_MTB_ was first assayed using *p*NP esters with various carbon chain lengths ranging from C2 to C16 (Figure 2B). Whatever their chain length, rLipG_MTB_ was able to hydrolyze all *p*NP esters, therefore displaying a large substrate specificity as previously reported by Rastogi et al. [21]. While similar S.A were obtained with short *p*NP-C4 and medium *p*NP-C8 esters (mean S.A = 4.26 ± 0.11 U/mg). However, rLipG_MTB_ exhibited a clear significant preference for *p*NP-C6 ester with a S.A value of 6.12 ± 0.17 U/mg. For *p*NP esters carrying more than eight carbon atoms, this S.A dropped sharply to 2.6 ± 0.18, 2.1 ± 0.21, 0.53 ± 0.08, and 0.19 ± 0.01 U/mg for *p*NP-C10, *p*NP-C12, *p*NP-C14, and *p*NP-C16, respectively (Figure 2B). Interestingly, amongst the Lip protein family member’s, rLipG_MTB_ substrate specificity using *p*NP esters was similar to those of the esterase’s LipU and LipR [23].

The lipase activity of rLipG_MTB_ has also been tested using a large panel of triacylglycerol (TAG) substrates, such as trioctanoin and triolein using the pH-stat technique, and pomegranate oil-coated TAG method [48]. In all cases, rLipG_MTB_ was not able to hydrolyze any of these TAGs, even when using highly sensitive fluorogenic TAG substrates [40]. It is well known that lipases differ from esterases by their ability to hydrolyze long-chain acylglycerols at the oil–water interface, whereas esterases can only hydrolyze substrates with short- or medium-fatty acid chains [61]. From these findings, rLipG_MTB_ could be considered as a true carboxylesterase, as recently proposed by Rastogi et al. [21].

Due to its genomic localization in the same cluster as the *mmaA* genes encoding for methyltransferase proteins, it is tempting to suggest that rLipG_MTB_ might also be able to hydrolyze the thioester bond of acyl-CoA or MA intermediates as previously shown for Cut6 (*Rv3802c*). Cut6 is an essential bifunctional phospholipase A and thioesterase enzyme belonging to the Cutinase-like family proteins from *M. tuberculosis* [62]. Thioesterases are a group of enzymes that catalyze the hydrolysis of a thioester bond between a carbonyl group and a sulphur atom. Here, the hydrolysis of palmitoyl-CoA was used to measure the thioesterase activity of rLipG_MTB_. As shown in Figure 2C, rLipG_MTB_ also exhibited a thioesterase activity with an S.A value of 2.10 ± 0.59 mU/mg. It is noteworthy that this value is of the same order of magnitude as that of TesA, a thioesterase of *M. tuberculosis* [42] with a S.A of 5.95 ± 0.85 mU/mg.

In order to investigate further rLipG_MTB_ enzyme activity, its phospholipase activity was also investigated. Interestingly, rLipG_MTB_ was able to hydrolyze only fluorescent phospholipids present at the *sn−1* stereospecific position with S.A value of 2.75 ± 0.25 mU/mg (Figure 2D) suggesting that rLipG_MTB_ is a strict PLA_1_. *T. lanuginosus* lipase PLA_1_ (TL PLA_1_) and *Bee venom* PLA_2_ (Bv PLA_2_) were used as positive controls for the PLA_1_ and PLA_2_ activities, respectively.

Finally, since Rastogi et al. [21] had proposed that the catalytic machinery was governed by Ser^123^, we thus generated by site-directed mutagenesis a rLipG_MTB_^S123A^ variant, in which this Ser^123^ residue was mutated into an Ala residue. As predicted, no enzyme activity was observed with fluorescent-phospholipids, *p*NP-substrates or palmitoyl-CoA with this inactive mutant (Figure 2E and data not shown). Taken together, these data provide full evidence that Ser^123^ contained in the consensus sequence G^121^A**S**MG^125^ is the catalytic residue responsible for the enzyme activity, and consequently, confirms that LipG_MTB_ is a true serine hydrolase. More specifically and given the level of S.A reached with the various substrates investigated, our results suggest that rLipG_MTB_ is a phospholipase A_1_ enzyme which also displays esterase and thioesterase activities.

### rLipG_MTB_ is inhibited by OX derivatives and CyC analogs

Two series of recently reported antitubercular inhibitors discovered in our laboratory (**OX** derivatives and **CyC** analogs), exhibit activity against enzymes by forming a covalent bond with the catalytic serine or cysteine residue in their active sites [27–29]. In order to further investigate the ability of these compounds to efficiently inhibit the lipolytic activity of rLipG_MTB_, six **OX** derivatives (i.e. **M*m*PPOX, iBPOX, iB*p*PPOX, HPOX, H*p*PPOX**, and **BePOX)** [27]; and five **CyC** analogs (i.e. the **CyC_17_, CyC_7α_, CyC_7β_, CyC_8α_**, and **CyC_8β_**) [28,29] were then tested for inhibition of rLipG_MTB_. In our previous work, whereas **iB*p*PPOX**, and **CyC_7β_** and **CyC_8α_** exhibited strong activity against both extracellular and intramacrophagic *M. tuberculosis*; **iBPOX** and **H*p*PPOX**, and **CyC_7α_** and **CyC_8β_** were found active on infected macrophages only [28,42]. In contrast, **HPOX** and **BePOX**, and **CyC_17_** efficiently block *M. tuberculosis* growth *in vitro* with no activity against intracellular bacilli [28,42]. **Orlistat** which has been previously reported as an inhibitor of LipG was used as positive control [63]. The chemical structures of the 12 compounds used in this study are provided in Supplementary Figure S2.

**Figure 3:**
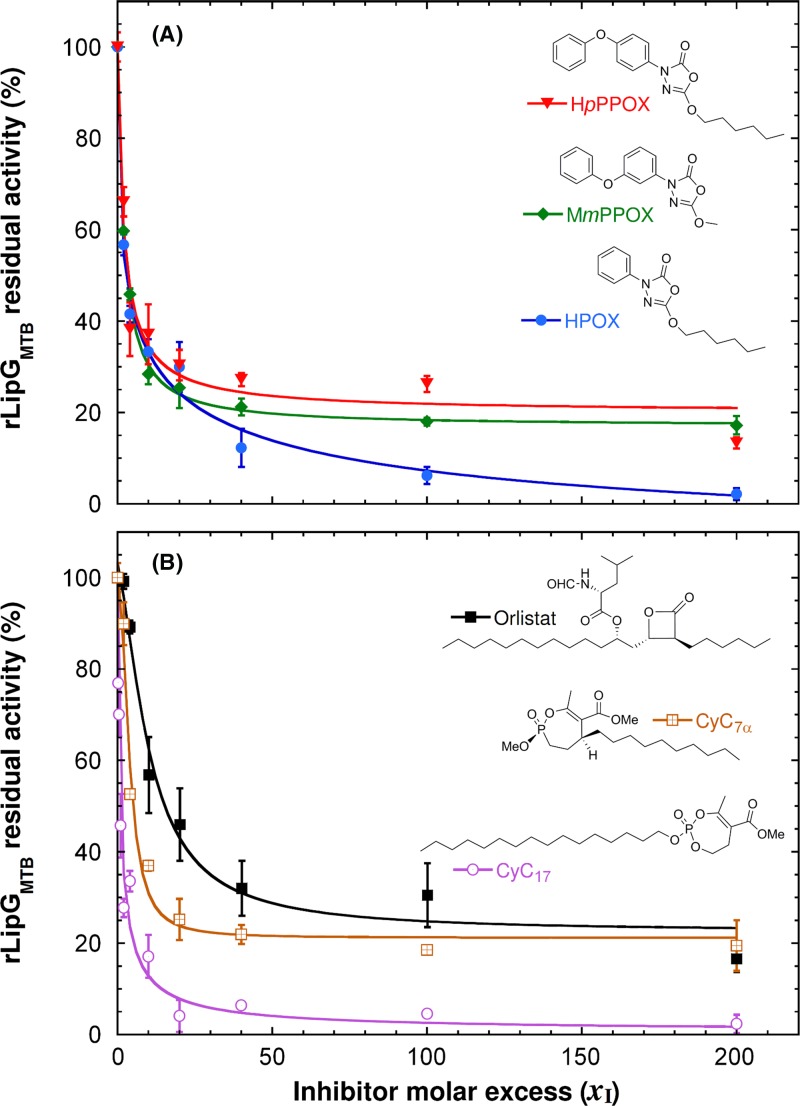
Inhibition studies of rLipG_MTB_ by OX derivatives and CyC analogs Residual activities of LipG_MTB_ measured on *p*NP-C6 as substrate. Effects of increasing molar excess (*x*_I_) of compounds (**A**) **M*m*PPOX, H*p*PPOX, HPOX**, and (**B**) **CyC_7_**_α_, **CyC_17_** and **Orlistat** on the rates of hydrolysis of *p*NP-C6. LipG_MTB_ was pre-incubated at various inhibitor molar excess (*x*_I_) for 30 min at 25°C. Kinetic assays were performed as described in **Experimental procedures** section. Results are expressed as mean values ± S.D. of three independent assays.

rLipG_MTB_ was inactivated by all the six **OX** derivatives, with medium 60.6% to good 93.8% inhibition levels at *x*_I_ = 100 (Table 2). The best inhibitors were **HPOX, M*m*PPOX**, and **H*p*PPOX**, which displayed inhibitor molar excess leading to 50% enzyme inhibition, i.e. *x*_I50_ values of ∼3.0 (Table 2 and Figure 3A). Amongst the **CyC** analogs, **CyC_7β_, CyC_8α_**, and **CyC_8β_** exhibited very weak inhibitory effects up to a high molar excess *x*_I_ = 100. Whereas, a strong dose-dependent inhibition was observed with **CyC_17_** and **CyC_7α_** (Table 2 and Figure 3B). In particular, **CyC_17_** was found to react almost stoichiometrically with rLipG_MTB_, as confirmed by its *x*_I50_ value of 0.98. It is noteworthy, that **Orlistat**, used as reference inhibitor, displayed only a moderate inhibitory activity against pure rLipG_MTB_ with only 69.5% inhibition at *x*_I_ = 100 and a *x*_I50_ value of 15.2, similar to those of **iB*p*PPOX, iBPOX**, and **BePOX**.

**Table 2:** Inhibition of rLipG_MTB_ after a 30-min incubation period with each inhibitor^1^

**Compounds**	% inhibition		x_I50_
	x_I_ = 4	x_I_ = 100	
**MmPPOX**	54.1 ± 1.5	82.0 ± 3.8	3.1
**iBpPPOX**	40.5 ± 3.1	60.6 ± 0.22	14.7
**iBPOX**	32.7 ± 0.70	80.3 ± 0.90	15.0
**HpPPOX**	61.8 ± 4.7	73.8 ± 1.0	3.5
**HPOX**	58.5 ± 1.4	93.8 ± 5.7	**2.9**
**BePOX**	9.8 ± 0.28	68.8 ± 1.1	12.5
**CyC_7α_**	47.4 ± 0.81	81.4 ± 1.6	5.0
**CyC_7β_**	7.9 ± 0.72	57.6 ± 2.0	48.1
**CyC_8α_**	2.5 ± 0.10	51.3 ± 2.6	58.5
**CyC_8β_**	3.2 ± 0.16	32.0 ± 1.5	>200
**CyC_17_**	66.4 ± 4.5	95.4 ± 2.6	**0.98**
**Orlistat**	10.8 ± 0.17	69.5 ± 5.3	15.2

1 Inhibition data (% of initial activity), at inhibitor molar excess (*x*_I_) of 4 and 100 related to 1 mol of enzyme. Results are expressed as mean values ± S.D. of at least three independent assays. The inhibitor molar excess leading to 50% lipase inhibition, *x*_I50_, was determined as described in **Experimental procedures** section.

### rLipG_MTB_ and rLipG_MS_ are peripheral membrane protein

**Figure 4:**
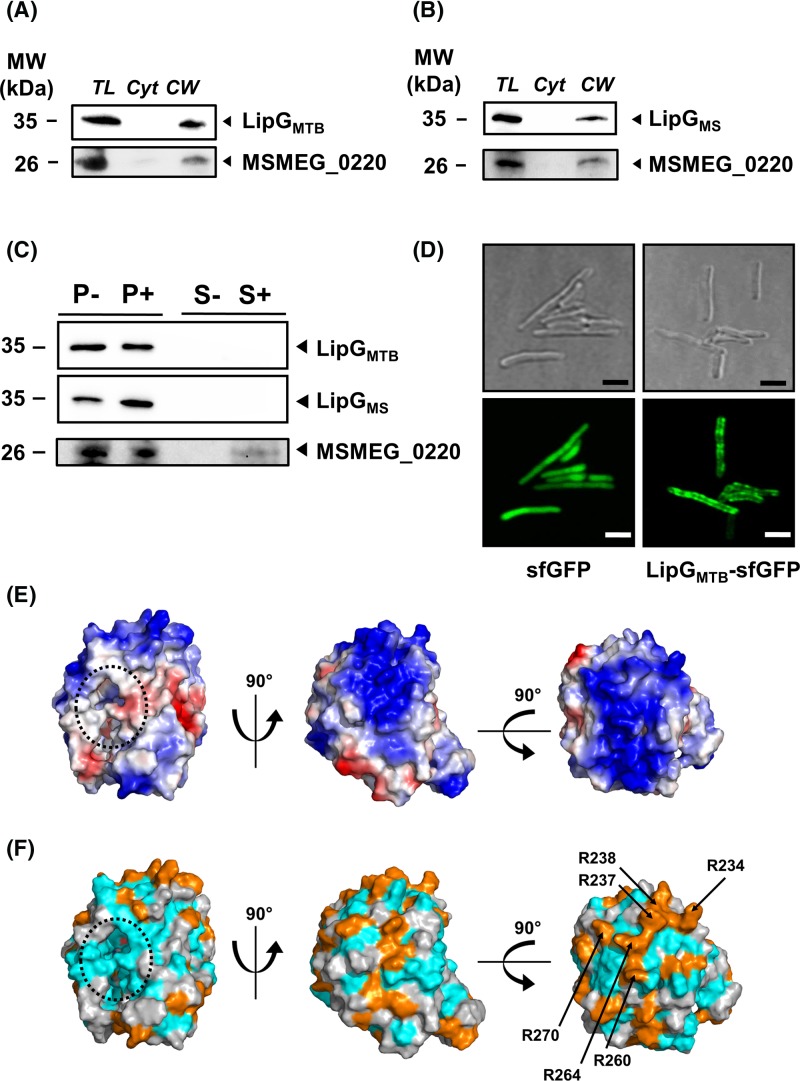
Subcellular location of LipG proteins (**A**,**B**) Subcellular localization of LipG_MS_ and LipG_MTB_ in *M. smegmatis groEL1ΔC* determined by ultracentrifugation. Recombinant cells expressing 6× His-tagged LipG were lysed, total lysate (TL), cytoplasm (Cyt), and cell wall (CW) fractions were separated by ultracentrifugation. Samples were loaded on to 12% SDS/PAGE and immunoblotted using HisProbe reagent. The exported MSMEG_0220 was used as control. (**C**) Determination of cell-surface-exposition of LipG_MS_ and LipG_MTB_ in *M. smegmatis groEL1ΔC* by detergent extraction. Recombinant cells expressing 6× His-tagged LipG were treated with PBS-buffer or with PBS-buffer containing Genapol®-X080 detergent. Pellet (P) and supernatant (S) fractions containing cytoplasmic and surface-exposed proteins respectively, were separated by centrifugation. Samples were loaded on to 12% SDS/PAGE and immunoblotted using HisProbe reagent. The exported MSMEG_0220 was used as a control. (**D**) Fluorescence microscopy analysis of *M. smegmatis groEL1ΔC* strains carrying either WT *sfGFP* gene or *lipG_MTB_-sfGFP* translational fusions. Cells were analyzed in both phase contrast (upper panel) and fluorescent channels (bottom panel). Scale bars represent 1 µm. (**E**) Electrostatic potential of LipG_MTB_ 3D model. The electrostatic surface potentials were displayed color-coded on to a van der Waals surface using the PyMOL Molecular Graphics System (version 1.8, Schrödinger, LLC). Red and blue colors represent net negative and positive charges, while white color represents overall neutral positions, respectively Black lines are showing the catalytic pocket. Two rotations of 90° were performed in order to provide a better view of the high potential area on top of the protein. (**F**) Position of positively charged residues in LipG_MTB_ 3D model. Positives residues were highlighted in orange. The two rotations of 90° were conserved in order to provide a better view of the positively charged residues on top of the protein. An arginine-rich patch has been identified and position of the respective residues (R^234^, R^237^, R^238^, R^260^, R^264^ and R^270^) are marked with black arrows.

Although LipG_MTB_ has been identified in the membrane fraction of *M. tuberculosis* H37Rv by using proteomic profiling approach [64], *in silico* experiments performed using SignalP 4.1 server [65] revealed that neither a peptide signal for the general secretion pathway or for the twin-arginine translocation pathway, nor a Type VII consensus secretion motif YxxxD/E were present in the primary sequence of LipG proteins. In this context, recombinant *M. smegmatis* strains, overexpressing either rLipG_MS_ (i.e. *MSMEG_1352* gene) or rLipG_MTB_ (i.e. *Rv0646c* gene) cloned under the control of the *hsp60* promoter in frame with a C-terminal 6× His-tag, were subjected to subcellular fractionation followed by immunoblotting experiments. The well-characterized cell wall exported monoglyceride lipase, MSMEG_0220, was used as control for cell fractionation [52,53]. In each case, both proteins were found only within the cell wall fraction and not in the soluble cytoplasmic fraction suggesting that LipG is a membrane/cell wall-associated protein (Figure 4A,B). Additional differential detergent extraction of surface-exposed mycobacterial proteins using Genapol®-X080 [54,55], followed by precipitation and immunoblotting (Figure 4C), demonstrated that LipG protein remains associated with the pellet fraction. This results suggest that this enzyme would not be accessible to the detergent and thus is not a surface-exposed protein. In contrast, MSMEG_0220 was found within the supernatant fraction, as expected (Figure 4C). All these data strengthen the fact that mycobacterial LipG are peripheral proteins, interacting with the inner leaflet of the cytoplasmic membrane.

To go further, the dynamic location of LipG within living cells was investigated by using fluorescence microscopy [66]. Gene fusions between *Rv0646c* or *MSMEG_1352* and the *sfGFP* gene encoding the sfGFP [67] were generated and used to transform *M. smegmatis* cells. Recombinant clones harboring stable fusions (Supplementary Figure S3) with a constant and important expression level were grown to mid-log exponential phase (i.e. OD_600_ = 1) in classical 7H9-S medium and further analyzed by fluorescence microscopy. As shown in Figure 4D (right panel), the strains harboring LipG_MTB_-sfGFP chimeric construct emitted a strong fluorescence signal mainly localized at the periphery of the inner mycobacterial cell membrane. In contrast, the fluorescence signal of the recombinant strain expressing only a wild-type sfGFP was less intense and diffuse through the cytoplasmic compartment (Figure 4D, left panel). Similar results were obtained when the bacteria was processed either at early exponential phase (i.e. OD_600_ = 0.2) or at the stationary phase (i.e. OD_600_ = 3). These findings strongly suggest that the resulting chimeric LipG proteins remain clustered into the bacterial cytoplasmic membrane whatever the phase of bacterial growth.

To get structural insights concerning LipG_MTB_ and its potential interaction within the cytoplasmic membrane, a 3D structural model was built using the I-TASSER server [35,36]. The X-ray structure of *Streptomyces lividans* methylesterase RdmC (PDB id: 1Q0R), solved at 1.45 Å resolution was used as a template [68]. This generated model revealed that LipG_MTB_ is a serine hydrolase constituted by 11 α-helices and 8 β-strands that are forming a central β-sheet (Supplementary Figure S4) as previously described [21]. A putative catalytic triad composed by Ser^123^, Asp^251^, and His^279^ is located within a highly accessible active site surrounded by hydrophobic residues forming the catalytic pocket (Supplementary Figure S4). In an effort to better characterize the molecular interactions that might govern LipG binding on to the inner cell membrane, the electrostatic surface potential of the previously built 3D model structure of LipG_MTB_ (Supplementary Figure S4) was generated and is displayed in Figure 4E,F. Interestingly, a positive electrostatic patch located opposite to the active site, on top of the model structure, was found. Notably, this specific amino-acid patch is mainly constituted by six arginine residues highly conserved in several LipG orthologs (Figure 4E,F and Supplementary Figure S5). Such arginine residues, which are indeed known to play important role in protein binding to zwitterionic and anionic glycerolipid polar head groups [69,70], might thus contribute to the binding of LipG to the cytoplasmic membrane (Figure 4E,F).

### Disruption of MSMEG_1352 triggers cell wall modifications

**Figure 5:**
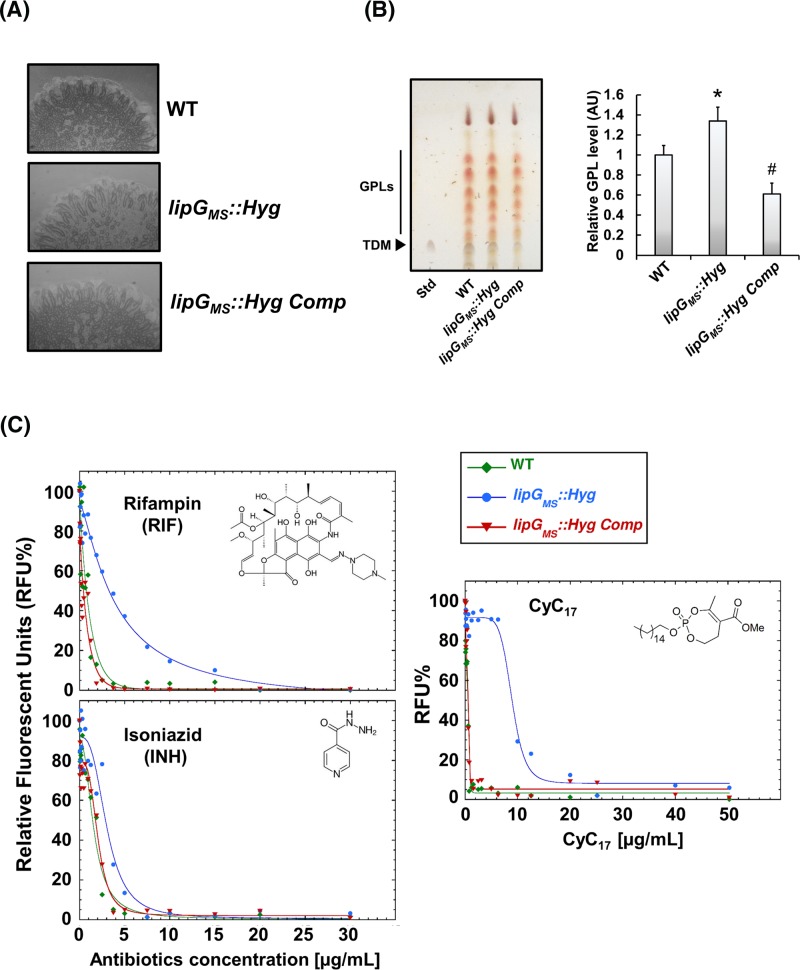
Disruption of *lipG_MS_* triggers cell-wall modifications and decreases antibiotics susceptibility (**A**) Colony morphology of WT, *lipG_MS_* disrupted and complemented strains. Recombinant strains were grown in 7H9-S before being spotted on to Middlebrook 7H11 agar plate devoid of Tween-80. Images are representative of two independent experiments. (**B**) GPL analysis of WT, *lipG_MS_* disrupted and complemented strains. Cells were grown in 7H9-S until OD_600 nm_ ∼1–1.5. Lipid extraction was followed by TLC (left panel) and densitometry analysis (right panel). Results are expressed as mean ± S.D. of total GPL level from three independent experiments, where WT level was arbitrarily adjusted to 1 relative unit. Statistical analysis was performed by using the Student’s *t* test. Relative GPL level from *lipG_MS_* disrupted mutant were compared with the WT where * corresponded to a *P*-value <0.05. Relative GPL level from *lipG_MS_* complemented strain were compared with the *lipG_MS_* disrupted mutant where # corresponded to a *P*-value <0.05. (**C**) Antibiotic susceptibility of WT, *lipG_MS_* disrupted and complemented strains. Susceptibility toward INH, RIF, and **CyC_17_** were determined using the REMA. Fluorometric MIC_50_s were determined by fitting the RFU% sigmoidal dose–response curves. Graph fitting are representative of three independent assays performed in duplicate.

In order to identify the physiological role of the LipG proteins within the mycobacterial lifecycle, a *M. smegmatis MSMEG_1352* disrupted strain named *lipG_MS_::Hyg*, in which the gene has been interrupted with a hygromycin resistance cassette, as well as a *lipG_MS_::Hyg* complemented strain (i.e. *lipG_MS_::Hyg Comp*) were produced (Supplementary Figure S6). When grown in 7H9-S medium for 48 h at 37°C, no significant differences in growth rate were observed between the WT, *lipG_MS_::Hyg*, and *lipG_MS_::Hyg Comp* strains. Interestingly, when the strains were grown on to 7H11TG agar plates (i.e. 7H11 medium supplemented with 0.02% Tween 80 and 0.2% glycerol), no differences in colony morphologies were observed with naked eyes. In contrast, when agar plates were spot-inoculated on to the same medium devoid of detergent and observed under a stereomicroscope, the *lipG_MS_::Hyg* mutant strain harbored a slightly smoother and rounder phenotype than the WT and *lipG_MS_::Hyg Comp* strains (Figure 5A). This finding would suggest that disruption of *lipG_MS_* may trigger some minor cell surface modifications (Figure 5A). TLC analysis of total lipid extract reveals that disruption of *lipG_MS_* induces a slight but significant overproduction of GPLs by 1.35-fold compared with WT strain (Figure 5B). In contrast, no detectable changes were observed within FAMEs and MAMEs levels as well as phospholipid content between each strain (Supplementary Figure S7A,B).

Although bacterial cell wall remodeling (including misregulation of GPL production) may also affect biofilm/pellicle formation, neither global nor visible modifications where observed amongst the WT, *lipG_MS_::Hyg*, and *lipG_MS_::Hyg Comp* strains regarding pellicle formation (Supplementary Figure S7C) in 7H9-S medium containing either glycerol or acetate as carbon source.

Since the mycobacterial cell wall is the first effective barrier against bactericidal compounds, we hypothesized that increase in GPLs could also play an important role in cell wall integrity. To answer this question, the WT, *lipG_MS_::Hyg*, and *lipG_MS_::Hyg Comp* strains were subjected to antibiotic susceptibility testing toward standard antibiotics INH and RIF, using the REMA method [28,59] (Figure 5C and Table 3). The effects of **Orlistat, HPOX** as well as the **CyC_17_** inhibitor on the growth of each strains were also investigated.

**Table 3:** Susceptibility testing of *M. smegmatis* WT and mutant strains

	MIC_50_ (µg/ml)^1^				Fold change compared with WT			
	INH	RIF	Orlistat	CyC_17_	INH	RIF	Orlistat	CyC_17_
WT	1.4	0.76	0.97	0.56	-	-	-	-
*lipG_MS_::Hyg*	2.9	3.3	6.5	9.6	×2.0	×4.3	×6.7	×15.7
*lipG_MS_::Hyg Comp*	1.8	0.89	1.2	0.60	×1.2	×1.2	×1.3	×0.94
*lipG_MS_::Hyg Comp_MTB_*	1.9	0.96	1.6	0.65	×1.3	×1.3	×1.6	×1.1

1 MIC_50_ values corresponding to the compound minimal concentration leading to 50% growth inhibition have been determined by the REMA method and are expressed as mean values of three independent assays performed in duplicate.

It is noteworthy that the respective MIC_50_ values obtained with INH (0.76 µg/ml), RIF (1.4 µg/ml), and **CyC_17_** (0.56 µg/ml) against the WT strain are in accordance with previously published studies (Figure 5C and Table 3) [28,29]. Regarding **Orlistat**, this well-known serine hydrolase inhibitor [23,71] is able to impair *M. smegmatis* growth with an MIC_50_ of 0.97 µg/ml. Surprisingly, deletion of *lipG_MS_* leads to an increased drug tolerance toward the four compounds. As depicted in Figure 5C and Table 3, MIC_50_ of INH, RIF, **Orlistat**, and **CyC_17_** against the *lipG_MS_::Hyg* strain amounted to 2.9, 3.3, 6.5, and 9.6 µg/ml, respectively, thus corresponding to a respective fold change compared with the WT strain of ×2.0, ×4.3, ×6.7, and ×15.7. The complementation of the mutant strain with the pVV16::*lipG_MS_* restored the drug susceptibility with mean MIC_50_ fold change compared with WT strain of approximately ×1.2 to ×1.3.

Similar results were obtained with **HPOX** inhibitor. MIC_50_ values of 8.4 and 18.4 µg/ml were obtained for the WT and *lipG_MS_::Hyg* strains, respectively; leading to a fold change compared with the WT strain of ×2.2. However, with this compound the complementation with pVV16::*lipG_MS_* vector only partially restored the drug susceptibility with an intermediate MIC_50_ value of 12.2 µg/ml (i.e. fold change compared with WT strain of approximately ×1.5).

Finally, we also carried out these experiments by performing complementation of the *lipG_MS_::Hyg* strain with the pVV16::*lipG_MTB_* vector (*lipG_MS_::Hyg Comp_MTB_*). Interestingly, production of LipG_MTB_ protein was perfectly able to restore the drug susceptibility, suggesting that the two proteins may possess similar enzymatic activities and/or physiological functions (Table 3).

To further confirm this result, the recombinant rLipG_MS_ was produced, purified, and biochemically characterized as described above for rLipG_MTB_ (Supplementary Figure S8 and Table S2). Both pure rLipG_MS_ and rLipG_MTB_ proteins thus exhibit the same substrate specificity toward *p*NP ester substrates, the best S.A being also found with *p*NP caproate (C6). Moreover, rLipG_MS_ was also able to hydrolyze acyl-CoA and phospholipids as substrates, confirming that the substrate specificity is well conserved between the two proteins. Despite their similar reactivity pattern with various esters, we noticed, however, that the rLipG_MS_ was more active than its ortholog (Supplementary Table S2). Interestingly, these differences where only observed *in vitro* using pure proteins, but not during *trans*-complementation with the *lipG_MTB_* gene during *in vivo* experiments as described above.

### Overexpression of lipG_MTB_ impacts free fatty acids and phospholipids homeostasis

**Figure 6:**
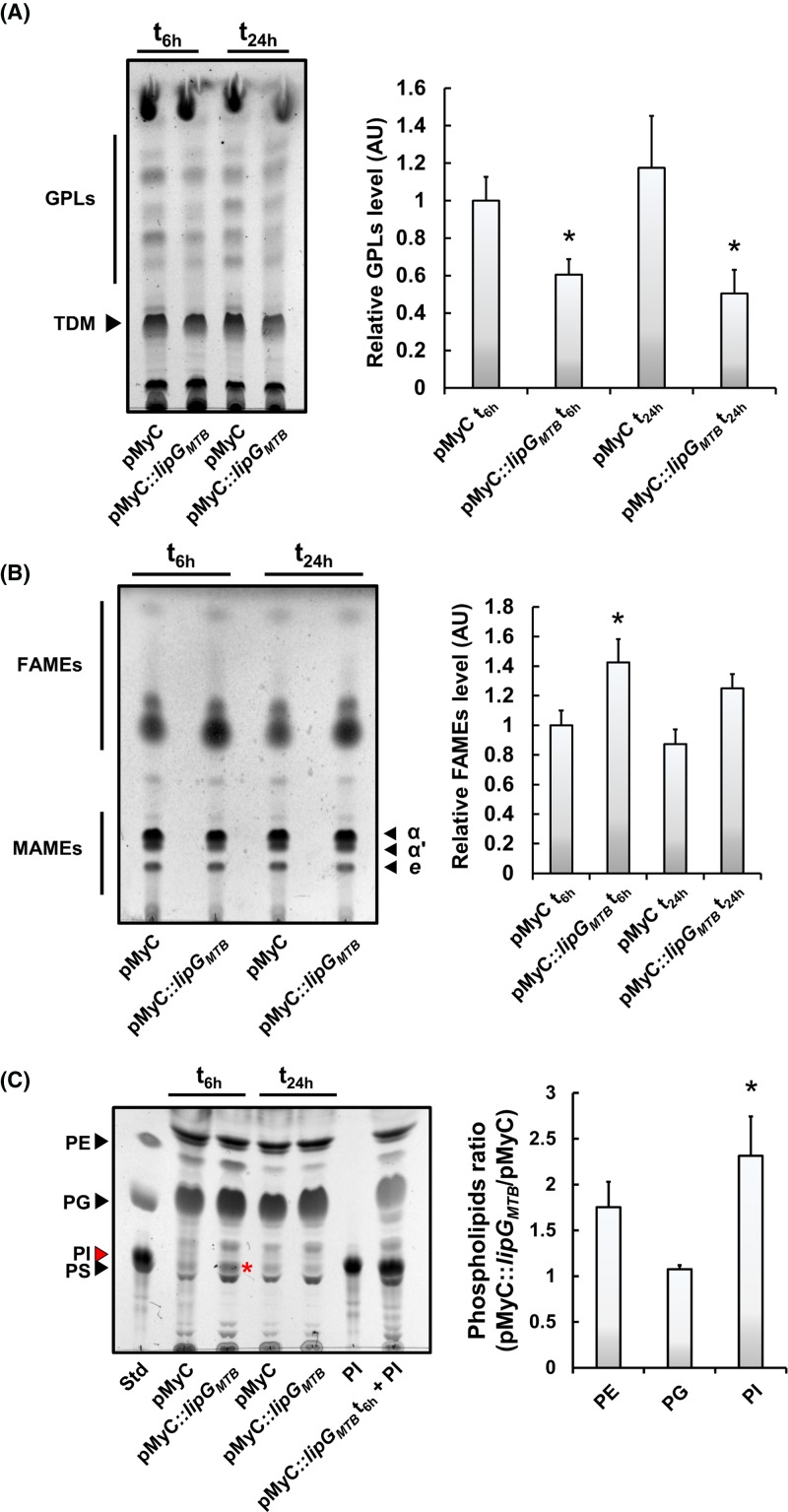
LipG_MTB_ impacts free fatty acids and phospholipids biosynthesis Mycobacterial cultures harboring pMyC and pMyC::*lipG_MTB_* were grown in 7H9-S medium until reaching OD_600 nm_ ∼0.5, then 0.2% of acetamide was added inside the culture medium. Cells were collected at 6 or 24 h post-induction corresponding to exponential or stationary phase, lyophilized and the same amount of dry cell weight was use for lipid extraction. After normalization, the same volume of each sample was loaded on to TLC. (**A**) Global GPL levels are decreasing in an *M. smegmatis* pMyC::*lipG_MTB_* overexpression strain. Lipid extracts of each samples were analyzed by TLC followed by densitometry analysis. (**B**) FAMEs levels are increasing in an *M. smegmatis* pMyC::*lipG_MTB_* overexpression strain. Lipid extracts of each samples were analyzed by TLC followed by densitometry analysis. In each case, results are expressed as mean ± S.D. of each respective total lipid species levels from at least two independent experiments, where control strain level at t_6 h_ was arbitrary adjusted to 1 relative unit. (**C**) Phospholipid biosynthesis is increased in an *M. smegmatis* pMyC::*lipG_MTB_* overexpression strain. Lipid extracts of each samples were analyzed by TLC followed by densitometry analysis. TLC was performed with CHCl_3_-MeOH-H_2_O (65:25:4; *v/v/v*) as eluent and PI overproduction is indicated with a red asterisk (left panel). Lipid sample from *M. smegmatis* pMyC::*lipG_MTB_* at t_6 h_ was also co-injected with pure PI standard as control lane, to clearly identify PI as an overproduced subspecies. Histograms correspond to densitometry analysis at t_6 h_. Results are displayed as intensity ratio where phospholipids levels from the pMyC::*lipG_MTB_* strain were divided by the phospholipids levels from the pMyC strain (right panel). Results are expressed as mean ± S.D. from three independent experiments. Statistical analysis was performed by using the Student’s *t* test where * correspond to a *P*-value <0.05. Abbreviation: PI, phosphatidylinositol.

Despite the observed impact of *lipG_MS_* gene disruption on to the penetration/diffusion of the latter antitubercular molecules within the mycobacterial envelope, no clear detectable phenotype has been observed. To overcome such issue, a recombinant strain harboring the pMyC::*lipG_MTB_* plasmid which allows a strong overproduction of the LipG_MTB_ protein upon acetamide induction, has been constructed. Approximately 6 and 24 h following induction by acetamide, total lipid extraction was performed on cell cultures either in exponential (OD_600_ ∼1–1.5) or stationary (OD_600_ ∼3–4) phase, respectively. The effect of LipG_MTB_ overproduction was scrutinized by TLC analysis. As expected from our previous results which showed a slight increase in GPL production in absence of LipG_MS_ (Figure 5A,B), in the strain overproducing LipG_MTB_ (Figure 6A), a clear drop in GPLs production was observed at 6 h (61 ± 8%) and 24 h (51 ± 12%) post-induction when compared with the WT strain harboring the empty pMyC vector as reference. However, in contrast with *M. smegmatis*, the fact that *M. tuberculosis* H_37_Rv does not produce GPLs may suggest a different functional role of LipG in this latter mycobacteria. This prompted us to further analyze other lipid subclasses. Accordingly, a semi-quantitative analysis of *M. smegmatis* strains harboring pMyC and pMyC::*lipG_MTB_* revealed that the overproduction of the protein significantly increases the level of FAMEs by 1.42-fold and 1.25-fold compared with control strain at 6 and 24 h post-induction, respectively (Figure 6B), without affecting MAMEs level or ratio.

Regarding phospholipid levels, surprisingly overexpression of *lipG_MTB_* slightly increases the phospholipid level of phosphatidylethanolamine (PE) and phosphatidylglycerol (PG) subspecies (1.75- and 1.07-fold in comparison with the control strain, respectively), but more importantly, this overproduction drastically impacts phosphatidylinositol (PI) production with a fold-change related to the control strain of ×2.3 (Figure 6C). These results were further confirmed by using 2D-TLC analysis, where a nice and efficient separation of all species of phospholipids allowed to confirm a consequent increase in PI biosynthesis upon LipG_MTB_ overproduction (Supplementary Figure S9).

Altogether, the increase in the levels of both FAMEs and specific subclass of phospholipids suggest that the LipG_MTB_ acyl-CoA-thioesterase activity may be the predominant biochemical activity *in vivo*, and that the products of the reaction are directly influencing cell wall phospholipids composition.

## Discussion

*M. tuberculosis* is one of the most successful infectious agents worldwide. Therefore, understanding the molecular mechanisms responsible for its pathogenesis remains one of the most important challenges in TB fundamental research. By using highly elaborate genetic screens, several independent teams have indeed tried to identify the genes required for *in vitro* or *in vivo* bacterial survival [26,72,73]. These approaches have highlighted the *Rv0646c* gene of *M. tuberculosis* which is required for adaptation and survival of the bacilli in mouse macrophages [26]. Interestingly, this gene has been annotated as encoding a putative lipase/esterase protein based on the presence of a G-x-S-x-G consensus pentapeptide motif. More importantly, the *Rv0646c* gene encoding LipG_MTB_ is widely conserved through the *Mycobacterium* genus and is associated with a gene cluster involved in MAs maturation processes [19]. Strikingly, bioinformatics analysis demonstrated that this gene is highly conserved in both slow- and fast-growing mycobacteria, and even in the obligate intracellular parasite *M. leprae*. This latter unusual pathogen is harboring a genome with a large number of inactivated pseudogenes which are not required anymore *in vivo* [74]. However, amongst the 24 genes encoding lipolytic enzymes from the Lip family in *M. tuberculosis*, only *lipE, lipU*, and *lipG* are conserved in *M. leprae*, thus reinforcing an essential role played by these enzymes in intracellular survival and proliferation [74,75]. In this study based on biochemical characterization of rLipG_MTB_ protein associated with microbiology experiments, we uncovered the potential role of this enzyme in mycobacterial lipid metabolism.

In accordance with a recent report, we confirmed that recombinant LipG_MTB_ protein is an esterase acting preferentially on medium chain-length substrates [21,75]. More interestingly, we also found that the protein was able to hydrolyze phospholipids at the *sn−1* position and cleave the thioester bond from palmitoyl-CoA, showing that rLipG_MTB_ possesses esterase and phospholipase, as well as thioesterase activities (Figure 2). Both phospholipase and thioesterase activities have been already observed by Parker et al. [62], who described that *M. tuberculosis Rv3802c* gene (*cut6*) encodes a functional phospholipase/thioesterase protein. Using radiolabeled phospholipids coupled with TLC analysis, the authors showed that Cut6 was not only able to act on PE, PC, and phosphatidylserine (PS) molecules [62], but that it was also able to hydrolyze both palmitoyl- and decanoyl-CoA, suggesting that the protein may act as an acyl donor *in vivo*.

The fact that LipG was required for adaptation and survival within macrophages, prompted us to investigate the inhibitory properties of two new series of serine hydrolase inhibitors active against *M. tuberculosis* growth [27–29]. During *in vitro* inhibition experiments on pure protein (Figure 3 and Table 2), the best inhibitors from the two series of compounds were **HPOX** (for the **OX** derivatives) on the one hand, and **CyC_17_** (for the **CyC** analogs) on the other hand; two molecules that are only active against extracellularly growing *M. tuberculosis* strain [27,28]. In the case of **CyC_17_**, this result was *a posteriori* predictable. Following activity-based protein profiling experiment coupled with proteomics analyses [28], LipG was indeed found as a potential target of this compound from a lysate of *M. tuberculosis* (unpublished data). However, its low coverage (21% sequence identity) with only five distinct peptide sequences identified together with a peptide score ∼2.0–3.4 (given by SequestHT algorithm) made that this protein was below our threshold and was not selected. Conversely, LipG was not identified from similar ABPP experiments performed with **HPOX** directly on living *M. tuberculosis* cells [27]. We recently demonstrated that both the **OX** and **CyC** families of compound are multitarget inhibitors, which are probably displaying pleiotropic and cumulative effects toward mycobacterial Ser- and Cys-containing enzymes involved in lipid metabolism and/or cell wall maintenance, thus leading to *M. tuberculosis* growth arrest [27,28]. From these findings and despite their strong inhibitory effect *in vitro* on pure LipG enzyme, we thus proposed that *in vivo* **HPOX** and **CyC_17_** preferentially impair other previously identified target enzymes such as the Ag85 complex or the thioesterase TesA rather than LipG_MTB_.

Based on proteomic analysis of the *M. tuberculosis* membrane fraction, it has been recently proposed that LipG_MTB_ may be localized to the cell surface and directly interact with the host immune system [21,64]. However, bioinformatics analysis did not reveal the presence of any secretion signals or specific motifs, which may indicate that the protein is surface exposed. Herein, using different cell-fractionation approaches, we demonstrated that LipG_MTB_ is associated with the membrane fraction [54,55,66], but not translocated across the cytoplasmic membrane and remains a cytoplasmic protein which is mainly peripheral (Figure 4). It has been described that such peripheral proteins require either a lipophilic attachment (i.e. acylation) or the presence of basic residues positively charged [69,70] which induce electrostatic interaction with the acidic and negatively charged phospholipids head-groups, thus allowing anchoring of the protein within the cytoplasmic membrane [70]. Based on our 3D structure model, surface electrostatic potential analysis allows identification of a region on top of the active site mainly composed by positively charged residues (Figure 4E,F). This area is essentially constituted by a C-terminal cluster of arginine residues fully conserved in numerous LipG proteins (Supplementary Figure S5). Since this type of electrostatic interaction has already been described for a mycobacterial protein involved in acyl-CoA metabolism [76], one can assume that this cluster of Arg residues would play an important role in LipG binding to the cell membrane, and thus for its physiological function.

In contrast with Cut6, which is known for being secreted and acting outside the mycobacterial cell [62,77], LipG_MTB_ and LipG_MS_ are presumably hydrolyzing cytoplasmic substrates. Two distinct strategies were employed: (*i*) the generation of a *lipG_MS_* disrupted strain which was *trans*-complemented with either *lipG_MS_* or *lipG_MTB_* and (*ii*) the generation of a *lipG_MTB_* heterologous overexpression strain which were useful in identifying the potential physiological role of LipG proteins and to define their substrates *in vivo*. On one hand, the observed increase in GPLs production due to *lipG* gene disruption (Figure 5) resulted in a significant reduced susceptibility to RIF, INH, **Orlistat, HPOX**, and above all **CyC_17_** for which the highest fold change compared with WT strain in MIC_50_ value has been reached (Table 3). Since RIF and INH are inhibiting specifically cytoplasmic targets, we hypothesized that such increase in GPL will affect cell wall fluidity and integrity; which subsequently impacts the penetration of the main two first-line anti-TB drugs [78]. Such results are in agreement with Khoo et al. work [79] who reported that *Mycobacterium avium* GPLs positive serovar were less sensitive to ethambutol than GPLs negative serovar, and concluded that hydrophobic GPLs production may alter cell wall permeability and correlate with antibiotic tolerance [79]. In other hand, the overproduction of the LipG_MTB_ protein leads to a significant increase in the FAMEs level (1.42-fold and 1.25-fold at 6 and 24 h post-induction, respectively), but more importantly a decrease in GPLs levels within *M. smegmatis* concomitant to an increase in level of phospholipid species and more particularly, the phosphatidyl inositol (Figure 6). However this is not the first time that such modifications of the cell wall composition have been observed. Similar phenotypes with drastic alteration of FAMEs/MAMEs and phospholipid subspecies levels have already been described in 2009, by Meniche et al. [58]*.* The authors demonstrated that overexpression of the *Corynebacterium glutamicum* gene *NCgl2775* encoding a functional thioesterase/phospholipase protein leads to an increase in MAs production concomitantly with a decrease in glycerophosphoslipids levels [58]. A similar result was also obtained when the MSMEG_6394 protein (i.e. NCgl2775 and Cut6 orthologs) was overproduced, which prompted the authors to propose that this kind of bifunctional enzyme is may be able to regulate membrane compositions under specific conditions [58].

Since not all the mycobacterial strain are GPLs producers, especially *M. tuberculosis* H_37_Rv, we postulated that this phenotype was more an indirect effect, than a direct catabolic process transposable to *M. tuberculosis*.

From all these findings, it is tempting to propose that LipG is mainly acting as a thioesterase dedicated to phospholipid metabolism *in vivo*. In a large number of bacteria, the contribution of thioesterase proteins to phospholipid biosynthetic process is however not well-defined. For example, in *E. coli*, glycerol starvation leads to an increase in acyl-ACP and a drastic diminution of phospholipid levels. Overexpression of TesA, a type I thioesterase, drastically reduced the accumulation of long-chain acyl-ACP species, probably by rerouting free fatty acids to phospholipids [80]. It has also been proposed that the *ybgC* gene encoding a functional acyl thioesterase is playing a role in phospholipid metabolism in Gram-negative bacteria [81–83]. Astonishingly, this latter enzyme is known for interacting with a large number of proteins dedicated to phospholipid homeostasis, such as the acyl-carrier protein, the glycerol 3-phosphate acyltransferase (PlsB), and the PS synthase (PssA) [82]. Such systems dedicated to phospholipids have not been described so far in mycobacteria, but a similar multicomponent complex is involved in MA biosynthesis [16,17]. Overall, it might be possible that some proteins work together in order to respond to specific stimuli and thus modulate biological membrane composition and fluidity. Such a physiological process has also been reported in eukaryotic cells, where upon inflammatory stimulus, the acyl-CoA thioesterase 7 (ACOT7) converts acyl-CoA molecules into free fatty acids, thus triggering a remodeling of phospholipid containing unsaturated long (≥C_20_)-acyl chains in macrophages [84].

Finally, PI is known for being an essential and abundant phospholipid species within mycobacterial cytoplasmic membrane [85], but also for being a major precursor for complex glycoconjugated lipids such as phosphatidyl mannosides, lipomannan, or lipoarabinomannan [86,87]. Observation of an important increase in PI subspecies during LipG_MTB_ overproduction may also impact the formation of such glycolipids that are highly contributing to virulence and modulation of the host immune response. The potential role of the LipG_MTB_ protein within these pathways is currently under investigation in our laboratory and may bring new insight concerning the biosynthesis of such complex lipids, but also the role of this highly conserved protein during host–pathogen cross-talk.

## Supporting information

**Table S1:** Primers used in this study. Restrictions sites or mutated codons are indicated in bold

**Table S2:** Specific activities (S.A) of rLipG proteins. Experiments were performed with same amount of rLipGMTB or rLipGMS following the procedures described in Material and Methods.

**Figure S1:** LipG proteins multiple sequence alignment

**Figure S2:** Chemical structure of CyC analogs and OX derivatives used in this study

**Figure S3:** Western-Blot analysis of recombinant strains expressing LipG or LipG-sfGFP translational fusions.

**Figure S4:** 3D model of LipGMTB protein and identification of a putative catalytic triad.

**Figure S5:** Arginine-rich patch conservation and composition

**Figure S6:** Schematic representation of *lipG_MS_::Hyg* strain construction and validation

**Figure S7:** Biofilm phenotypes and lipid analysis of WT, *lipG_MS_::Hyg* and complemented strains

**Figure S8:** Purification and biochemical characterization of recombinant rLipG_MS_

**Figure S9:** Phosphatidylinositol identification by two-dimension TLC analysis

## Abbreviations

CyC: cyclophostin and cyclipostins analogs
DTNB: 5-5′-Dithio-bis 2-nitrobenzoic acid
FAME: fatty acid methyl ester
FAS: fatty acid synthase
GPL: glycopeptidolipid
INH: isoniazid
MA: mycolic acid
MAME: MA methyl ester
OX: oxadiazolone-core derivative
palmitoyl-CoA: palmitoyl-Coenzyme A
PI: phosphatidylinositol
*p*NP: *p*-nitrophenyl
PS: phosphatidylserine
REMA: resazurin microtiter assay
RIF: rifampicin
S.A: specific activity
sfGFP: superfolder GFP
TAG: triacylglycerol
TB: tuberculosis
