# Supplementary material for: LipG a bifunctional phospholipase/thioesterase involved in mycobacterial envelope remodeling
Source: Biosci Rep. 2018 Dec 18;38(6):BSR20181953. doi: 10.1042/BSR20181953 (PMC6435540; doi:10.1042/BSR20181953)
Supplement: Supplementary file 1 [file bsr20181953_Supp1.pdf]

Supplementary material

LipG a bifunctional phospholipase/thioesterase involved in mycobacterial envelope remodeling

Pierre Santucci[1], Vanessa Point[1], Isabelle Poncin[1], Alexandre Guy[2], Céline Crauste[2], Carole Serveau-Avesque[3], Jean Marie Galano[2], Chistopher D. Spilling[4], Jean-François Cavalier[1] and Stéphane Canaan[1,*]

[1] Aix-Marseille Univ, CNRS, LISM, IMM FR3479, Marseille, France.

[2] Institut des Biomolécules Max Mousseron (IBMM), UMR 5247, Université de Montpellier, CNRS, ENSCM, 15 Avenue Charles Flahault, 34093 Montpellier Cedex 5, France

[3] Aix-Marseille Univ, CNRS, Centrale Marseille, ISM2, Marseille, France.

[4] Department of Chemistry and Biochemistry, University of Missouri, One University Boulevard, St. Louis, MO 63121, USA

Short title: Biochemical characterization and physiological role of LipG

Keywords: *Mycobacterium tuberculosis*, lipolytic enzymes, phospholipid homeostasis, cell-envelope, antibiotics

*** Corresponding author**: canaan@imm.cnrs.fr (S. Canaan)

Table S1. Primers used in this study. Restrictions sites or mutated codons are indicated in bold

Primer Numbers	***Name***	***Sequence (5'-3')***
$P1_{MTB}$	*Rv0646c*-attB1 F	ggggacaagtttgtacaaaaaagcaggcttcgaaggagatagaaccatgcatcaccatcacca tcacgaaaacctgtacttccagggtgacatccgtagcgg
$P2_{MTB}$	*Rv0646c*-attB2 R	ggggaccactttgtacaagaaagctgggtctcagccggcctcagagaagttg
$P1_{MS}$	*MSMEG_1352*-attB1 F	ggggacaagtttgtacaaaaaagcaggcttcgaaggagatagaaccatgcatcaccatcacca tcacgaaaacctgtacttccagggtcagacccgcaccgggctcgcg
$P2_{MS}$	*MSMEG_1352*-attB2 R	ggggaccactttgtacaagaaagctgggtctcaacccaccacggcaaagtttg
P3	*Rv0646c* S123A F	gtcgtcggggcg**gcg**atgggcggcatg
P4	*Rv0646c* S123A R	catgccgcccat**cgc**cgccccgacgac
P5	*Rv0646c* NdeI F	ggaattc**catatg**gacatccgtagcggtggacatc (Nde1)
P6	*Rv0646c* BamHI R	gcagct**ggatcc**ctgagccggcctcagagaagttgc (BamH1)
P7	*MSMEG_1352* NdeI F	cttc**catatg**cagacccgcaccgggc (Nde1)
P8	*MSMEG_1352* BamHI R	agct**ggatcc**ctgaacccaccacggcaaagtttg (BamH1)
P9	*MSMEG_1352* FL500 F	ttcgacttcggcatcatgggccgc
P10	*MSMEG_1352* FL500 R	ctcgacgtgcgcctgctgattgcggc
P11	*MSMEG_1352* FL600 F	gctgttcagcgtgttcgagggc
P12	*MSMEG_1352* FL600 R	ctccgatcgacacgatgcgg
P13	*Sf-GFP* EcoRI F	gcaagcggcgactgacgct**gaattc**agcaaaggagaagaacttttcac (EcoR1)
P14	*Sf-GFP* HindIII F	tatat**aagctt**agcaaaggagaagaacttttc (HindIII)
P15	*Sf-GFP* HindIII R	gtggtg**aagctt**cagtttgtatagttcatccatgcc (HindIII)
P16	*HygR* F	**cccggg**taccaagccctc (Sma1)
P17	*HygR* R	**cccggg**ggcgtcagg (Sma1)

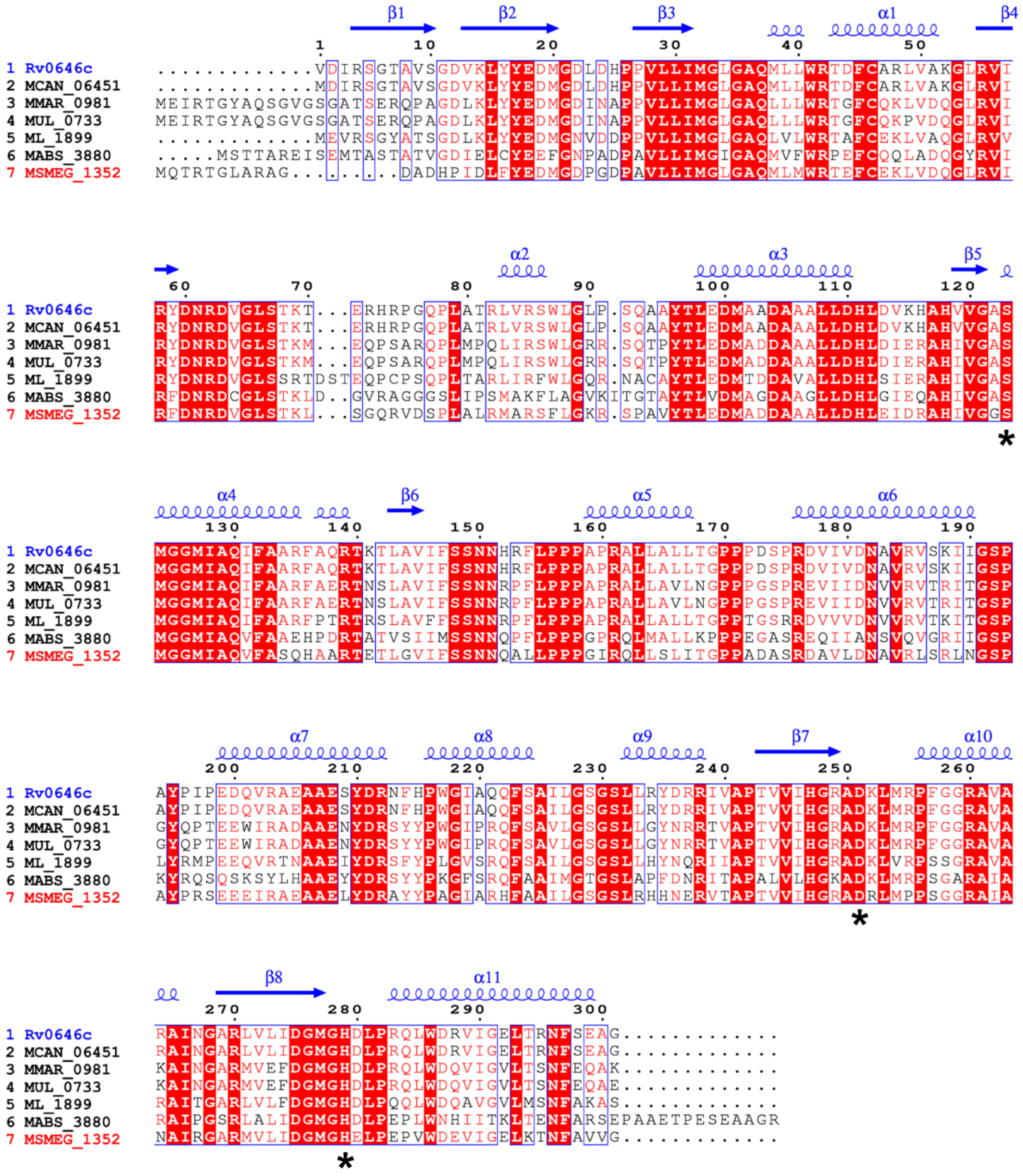

Figure S1: LipG proteins multiple sequence alignment

LipG sequences from *M. tuberculosis* (Rv0646c), *M. canettii* (MCAN_06451) *M. marinum* (MMAR_0981), *M. ulcerans* (MUL_0733), *M. leprae* (ML_1899), *M. abscessus* (MABS_3880) and *M. smegmatis* (MSMEG_1352) were obtained from Mycobrowser and NCBI database. Alignment was performed with Multalin software and displayed using ESPrit 3.0 software. Secondary structure information (displayed in blue) were obtained from the 3D model of $LipG_{MTB}$ generated using I-TASSER server. Residues from the catalytic triad are indicated with black stars.

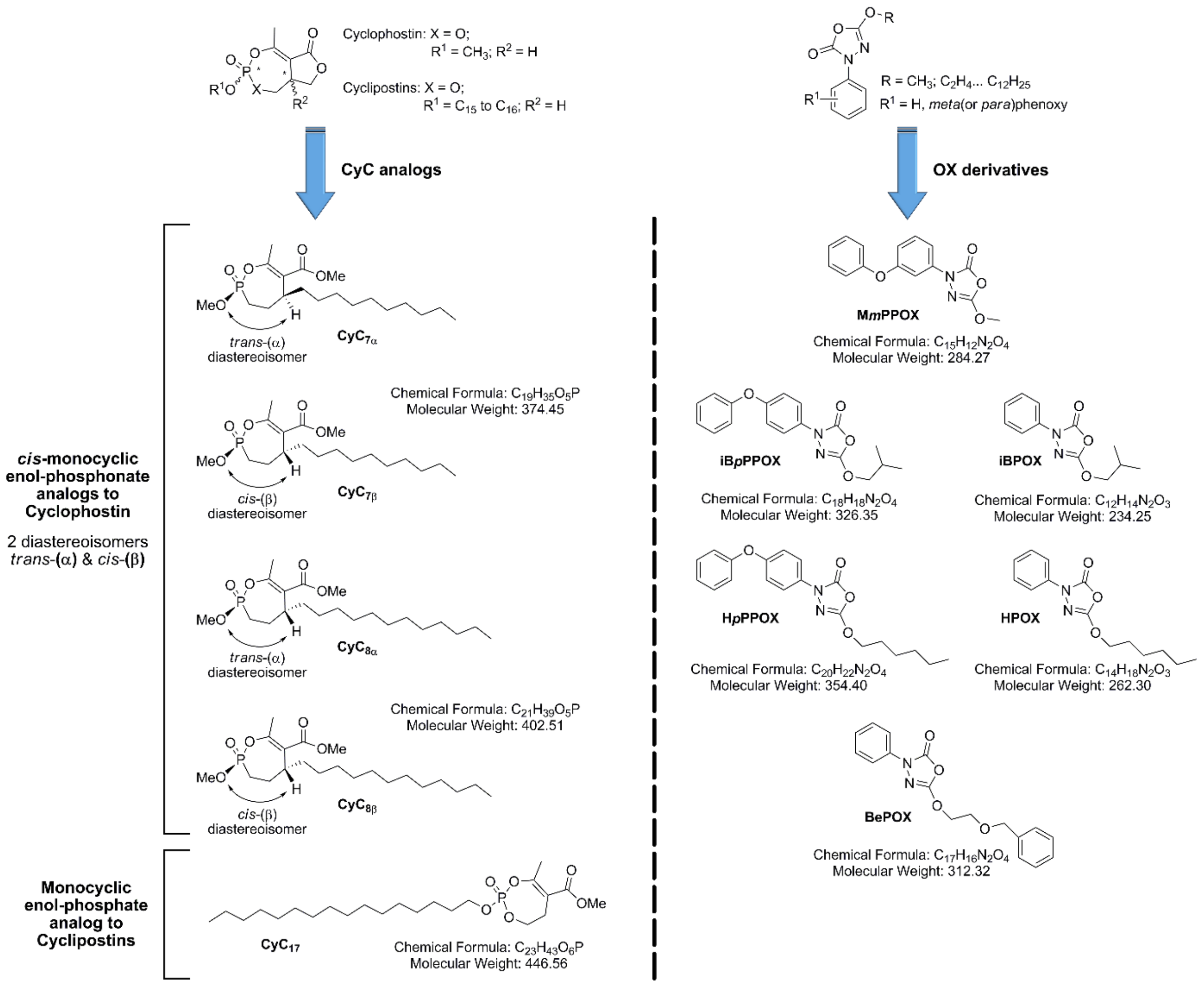

Figure S2: Chemical structure of CyC analogs and OX derivatives used in this study

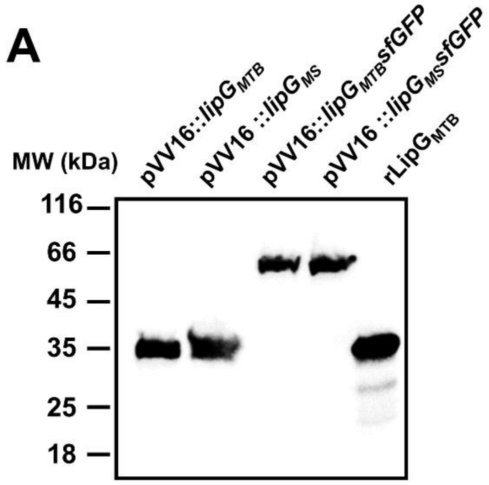

Figure S3: Western-Blot analysis of recombinant strains expressing LipG or LipG-sfGFP translational fusions.

Recombinants *M. smegmatis groEL1ΔC* cells producing LipG$_{(MTB,\ MS)}$ or LipG$_{(MTB,\ MS)}$-sfGFP translational fusions were lysed and total lysates were loaded onto Any kD Stain Free Gel (Biorad) followed by immunoblotting experiment using HisProbe reagent. Approximately 200 ng of rLipG$_{MTB}$ purified from *E. coli* was used as positive control.

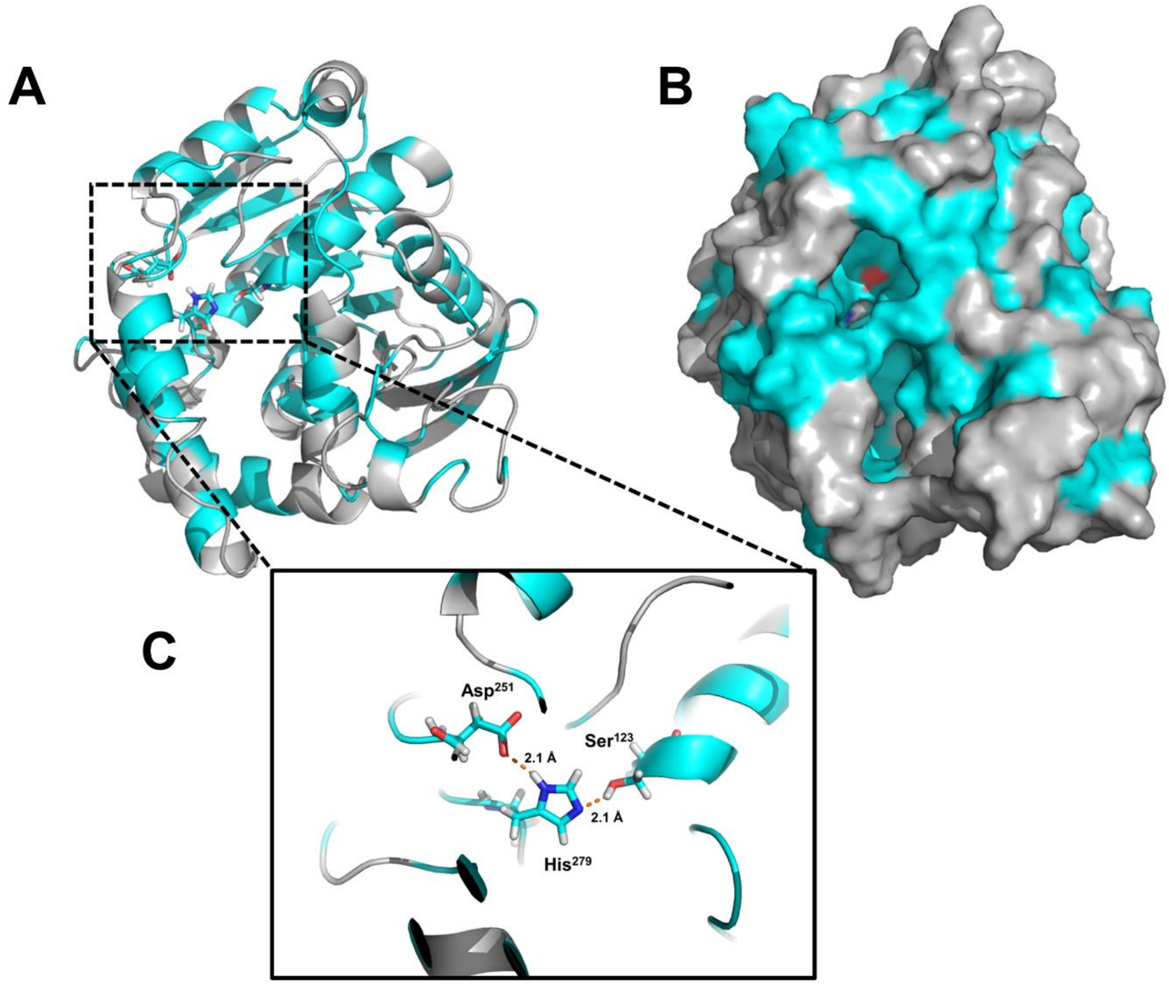

Figure S4: 3D model of LipG$_{MTB}$ protein and identification of a putative catalytic triad. The model was generated using the I-TASSER server and the X-ray structure of *Streptomyces lividans* methylesterase RdmC (PDB id: 1Q0R), solved at 1.45 Å resolution was used as template. The model was displayed using the PyMOL Molecular Graphics System (version 1.8, Schrödinger, LLC). Hydrophobic residues were colored in cyan whereas the other residues where colored in gray. **(A)** 3D model of LipG$_{MTB}$ protein in cartoon representation where the putative catalytic triad (Ser^{123}, Asp^{251} and His^{279}) is located within a hydrophobic pocket. **(B)** Surface representation of the 3D model of LipG$_{MTB}$ showing a highly accessible active site surrounded by hydrophobic residues forming the catalytic pocket. **(C)** Zoom of the catalytic pocket showing the orientation and the distance between the putative catalytic residues.

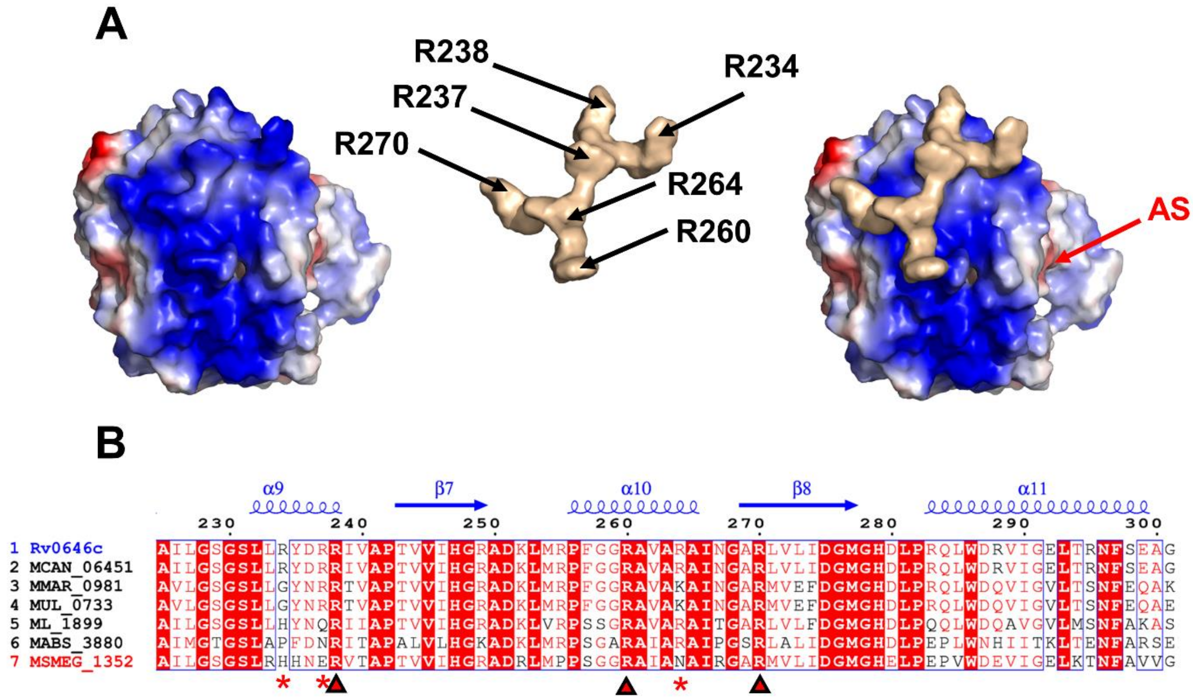

Figure S5: Arginine-rich patch conservation and composition

(A) Electrostatic potential of $LipG_{MTB}$ 3D model and position of the 6×Arg patch. The electrostatic surface potentials were displayed color-coded onto a van der Waals surface using the PyMOL Molecular Graphics System (version 1.8, Schrödinger, LLC). Red and blue colors represent negative and positive charges, while white color represents overall neutral positions, respectively. Here is the high potential area on top of the protein where the location of active site (AS) is indicated with a red arrow. The gold region is representing the position of the respective residues (R^{234}, R^{237}, R^{238}, R^{260}, R^{264} and R^{270}) which constitute a positively charged patch. **(B)** LipG multiple sequence alignment generated in **Figure S1** has been used to depict the position and conservation of the Arg-rich patch in LipG proteins. Strictly conserved Arg residues were highlighted with a red arrow whereas other Arg residues contained in the patch were highlighted with red stars.

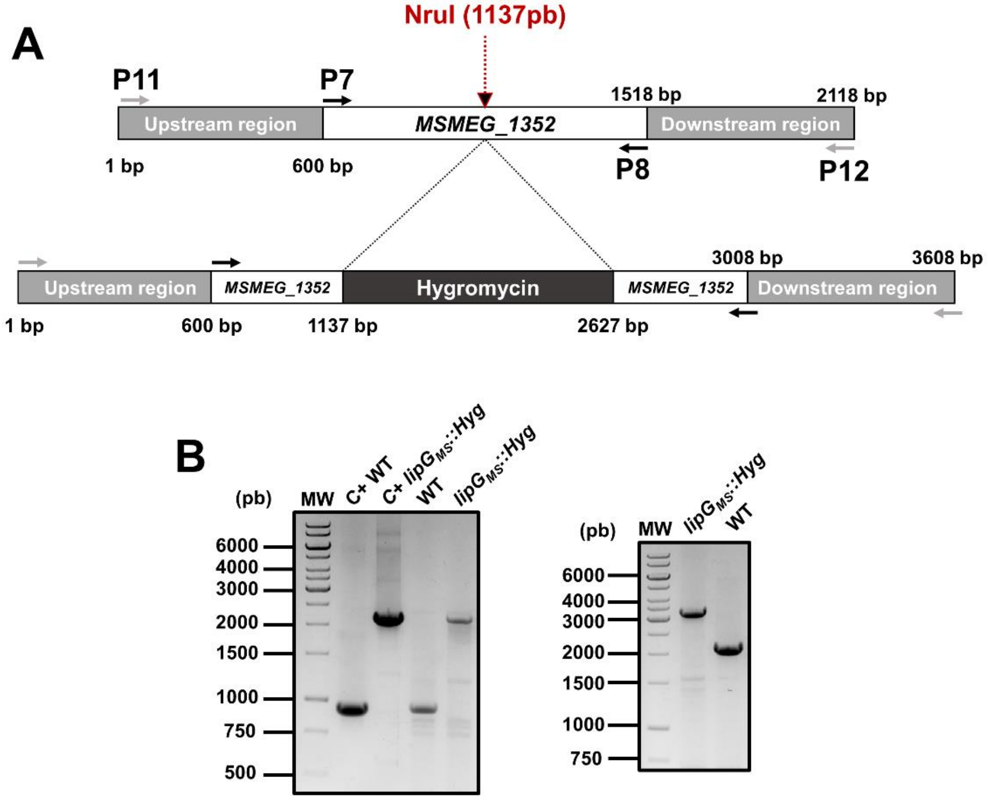

Figure S6: Schematic representation of *lipG*$_{MS}$*::Hyg* strain construction and validation
(A) Schematic representation of *lipG*$_{MS}$ (*MSMEG_1352*) genetic locus and construction of the *lipG*$_{MS}$*::Hyg* strain. **(B)** Genetic profiles of both WT and *lipG*$_{MS}$*::Hyg* strain analyzed by PCR. Left panel corresponds to PCR amplifications from WT and *lipG*$_{MS}$*::Hyg* with P7 and P8 primers. C+ WT and C+ *lipG*$_{MS}$*::Hyg* correspond to control amplification from pJET-*lipG*$_{MS}$ and pJET-*lipG*$_{MS}$*::Hyg* respectively. Right panel corresponds to PCR amplifications from WT and *lipG*$_{MS}$*::Hyg* with P11 and P12 primers thus demonstrating that the insertion of the hygromycin cassette occurs at the desired locus.

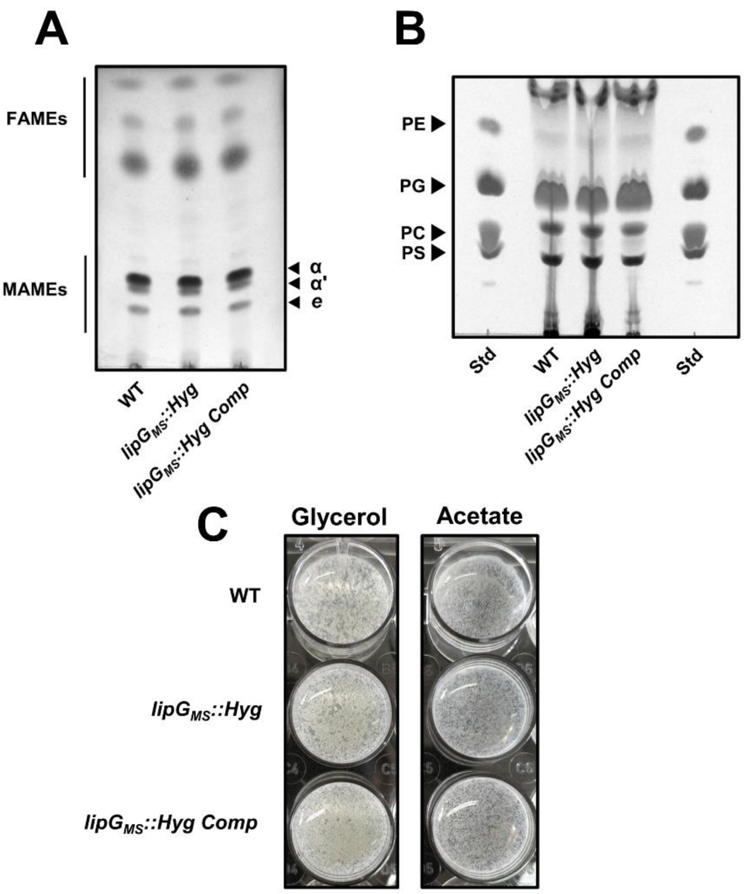

Figure S7: Biofilm phenotypes and lipid analysis of WT, *lipG$_{MS}$::Hyg* and complemented strains

(A-B) *lipG$_{MS}$* disruption does not affect FAMES, MAMES and phospholipids levels. Mycobacterial cultures (WT, *lipG$_{MS}$::Hyg* and *lipG$_{MS}$::Hyg* Comp strains) were grown in 7H9-S medium until reaching OD_{600nm}~1-1.5. Cells were collected, lyophilized and the same amount of dry cell weight was use for lipid extraction. In all cases, lipid extracts of each samples were analyzed by TLC followed by densitometry analysis. **(A)** TLC analysis of FAMEs and MAMEs. **(B)** TLC analysis of phospholipid subspecies. **(C)** Biofilm formation in absence or presence of *lipG$_{MS}$* is not affected. Cells were grown in 7H9-S medium until reaching OD_{600nm}~1-1.5. These sub-cultures were used to inoculate a 24-well tissue culture plate containing 7H9 medium devoid of detergent, supplemented with either 0.2% of glycerol or 0.2% acetate as sole carbon source. Plates were sealed and incubated at 37°C without shaking for 7 days.

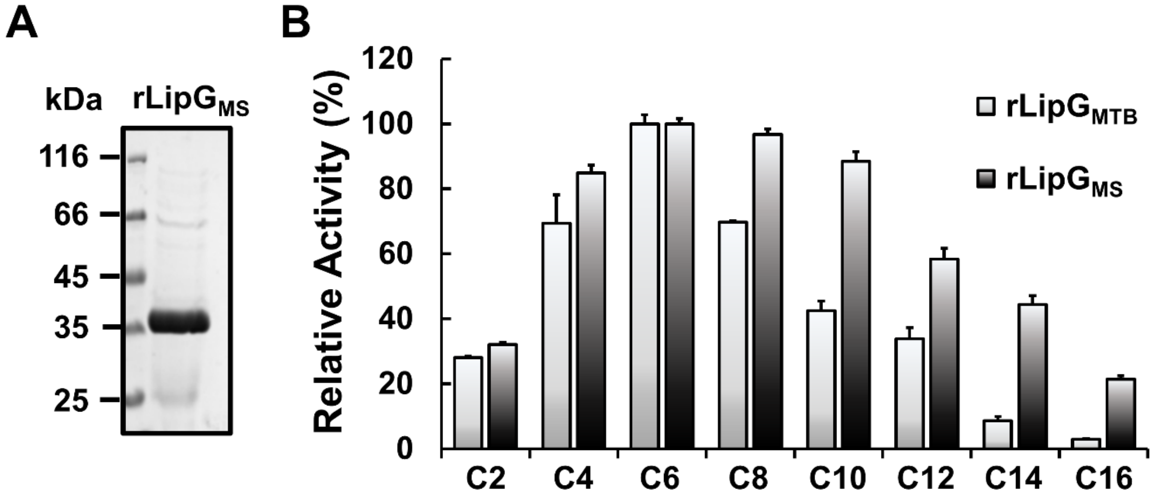

Figure S8: Purification and biochemical characterization of recombinant rLipG$_{MS}$
(A) Purity assessment of rLipG$_{MS}$. rLipG$_{MS}$ (10µg) was loaded onto 12% polyacrylamide gel. Unstained protein molecular weight marker was used as standard (Euromedex). **(B)** Carboxylesterase activities determination on *p*NP-ester substrates with chain lengths varying from C2 to C16 as described in Material and Methods. Substrate specificity of both rLipG$_{MTB}$ and rLipG$_{MS}$ towards *p*NP-esters of different chain length. Specific activity determined from *p*NP-C6 hydrolysis was used as standard, and results are expressed in relative activity (%).

Table S2. Specific activities (S.A) of rLipG proteins. Experiments were performed with same amount of rLipG$_{MTB}$ or rLipG$_{MS}$ following the procedures described in **Material and Methods**.

**Substrates**	**LipG$_{MTB}$**	**LipG$_{MS}$**	**Fold Change (LipG$_{MS}$/LipG$_{MTB}$)**
	**Specific activity (S.A) U/mg**		
***p*-nitrophenyl esters** (at 37°C)			
*p*NP acetate (C2)	1.72 ±0.03	8.88 ±0.17	×5.2
*p*NP butyrate (C4)	4.25 ±0.53	23.5 ±0.67	×5.5
*p*NP caproate (C6)	6.12 ±0.17	27.7 ±0.44	×4.5
*p*NP caprylate (C8)	4.27 ±0.23	26.8 ±0.44	×6.3
*p*NP caprate (C10)	2.60 ±0.18	24.5 ±0.79	×9.4
*p*NP laurate (C12)	2.07 ±0.21	16.2 ±0.90	×7.8
*p*NP myristate (C14)	0.53 ±0.08	12.3 ±0.76	×23.2
*p*NP palmitate (C16)	0.18 ±0.01	5.90 ±0.29	×32.9
	**Specific activity (S.A) mU/mg**		
**Acyl-CoA** (at 37°C)			
Palmitoyl-CoA (C16)	2.10 ±0.59	3.88 ±0.26	×1.85
**Phospholipids** (at 25°C)			
Fluorogenic PLA$_1$ specific substrate	2.74 ±0.24	2.39 ±0.25	×0.87

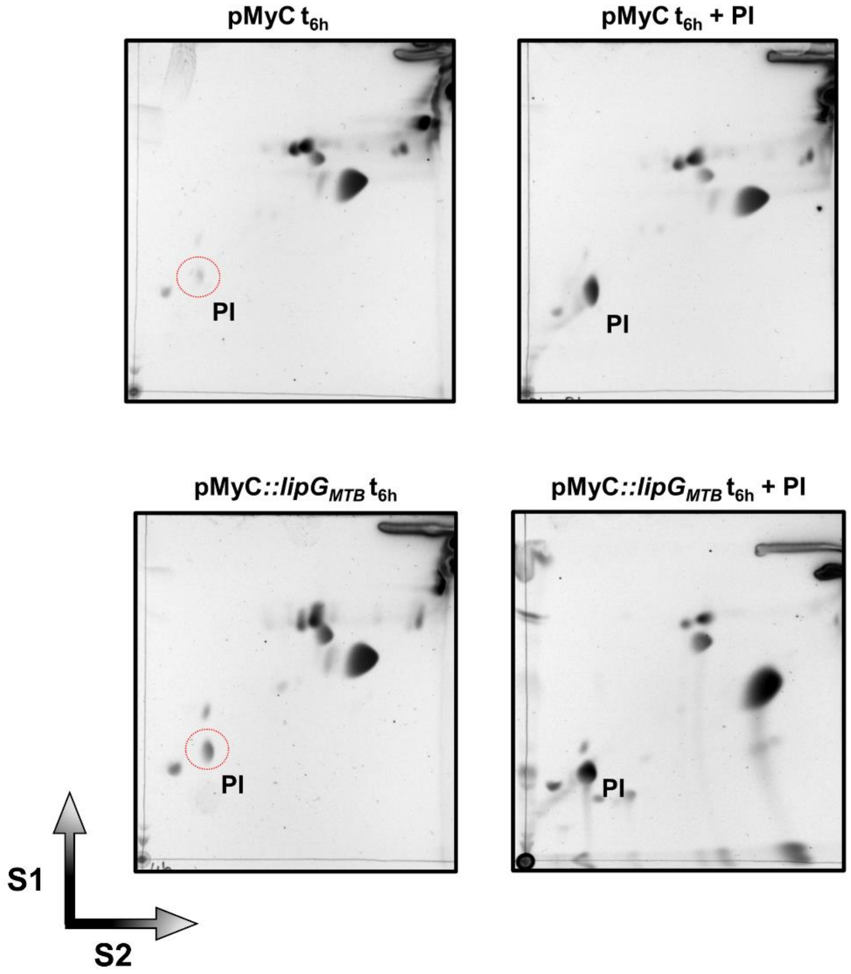

Figure S9: Phosphatidylinositol identification by two-dimension TLC analysis
Phosphatidylinositol (PI) biosynthesis is increased in *M. smegmatis* pMyC::*lipG$_{MTB}$* overexpression strain. Lipid extracts from mycobacterial cultures harboring pMyC t_{6h} and pMyC::*lipG$_{MTB}$* t_{6h} were analyzed by 2D-TLC. TLC were first resolved with $CHCl_3$-MeOH-H_2O (65:25:4; *v*/*v*/*v*) as solvent system 1 (S1) and $CHCl_3$-MeOH-CH_3COOH-H_2O (80:12:15:3; *v*/*v*/*v*/*v*) as solvent system 2 (S2). Revelation was performed as described in the Experimental procedures section. To further identify PI and confirm its overproduction in *M. smegmatis* pMyC::*lipG$_{MTB}$* strain, TLC analysis were repeated by co-injecting the samples with pure PI standard (right panels). Based on these observations, PI spots have been annotated in black and indicated with red circles.
